# Sagan Dalya Tea, a New “Old” Probable Adaptogenic Drug: Metabolic Characterization and Bioactivity Potentials of *Rhododendron adamsii* Leaves

**DOI:** 10.3390/antiox10060863

**Published:** 2021-05-27

**Authors:** Daniil N. Olennikov, Vyacheslav M. Nikolaev, Nadezhda K. Chirikova

**Affiliations:** 1 Laboratory of Medical and Biological Research, Institute of General and Experimental Biology, Siberian Division, Russian Academy of Science, 670047 Ulan-Ude, Russia; 2 Department of the Adaptation Mechanisms Study, Yakutsk Scientific Center of Complex Medical Problems, 677000 Yakutsk, Russia; nikolaev1126@mail.ru; 3 Department of Biology, Institute of Natural Sciences, North-Eastern Federal University, 677027 Yakutsk, Russia; hofnung@mail.ru

**Keywords:** *Rhododendron adamsii*, Sagan Dalya, liquid chromatography-mass spectrometry, seasonal variation, antioxidant activity, adaptogen, swimming to exhaustion test

## Abstract

Adams’ rhododendron (*Rhododendron adamsii* Rehder) or Sagan Dalya tea is a famous Siberian evergreen medical plant of the Ericaceae family used in traditional medicines of Buryats, Yakuts, and Mongols as a tonic, stimulant, and adaptogenic drug. The high popularity of *R. adamsii* coupled with poor scientific knowledge prompted the addressing of gaps related to metabolic and biomedical data of Sagan Dalya tea. The application of solid-phase extraction and liquid chromatography-mass spectrometric techniques for the metabolomic study of *R. adamsii* leaf extracts resulted in the identification of more than 170 compounds, including carbohydrates, organic acids, simple phenol glycosides, triterpene glycosides, flavonoids, prenylated phenols, benzoic acid derivatives, hydroxycinnamates, dihydrochalcones, catechins, and procyanidins, most of which were identified for the first time in the plant. Extended surveys of the seasonal content of all detected compounds prove that specific metabolite variations reflect the bioactivity of *R. adamsii* extracts. Regarding in vitro methods, the expressed antioxidant potential of *R. adamsii* extracts was investigated via radical-scavenging, nitric oxide scavenging, and ferrous (II) ion chelating assays. The animal-based swimming to exhaustion test demonstrates the stimulating influence of *R. adamsii* extract on physical performance and endurance, concluding that the drug could act as an adaptogen. Thus, Sagan Dalya tea (*R. adamsii*) has confirmed its “old” application as a tonic remedy and requires further precise study as a novel adaptogenic plant.

## 1. Introduction

Modern changes to human life, as well as the harmful effects of the environment observed in our time, often lead to a sharp decrease in the body’s adaptive capacity and functional reserves. Therefore, the study of the mechanisms underlying the adaptation process and the search for new drugs and ways to increase the body’s functional reserves are among the main aims of modern biomedical sciences. To increase the body’s resistance to adverse factors, drugs of various groups are used, the most universal of which are natural adaptogens, which increase the body’s performance and transfer it into a state of nonspecific increased resistance [1]. The relative safety and breadth of the therapeutic action of these natural remedies make them especially valuable for increasing the performance of people in unusual climatic conditions with static and dynamic industrial overloads, professional athletes, and the elderly, including those suffering from chronic diseases, alongside increasing the performance and mental activity of all people [2]. The effects of adaptogens on the human body are multi-faceted: adaptogens exhibit immunostimulating activity and improve anabolism, stimulate the central nervous and endocrine systems, modulate the sensitivity of cell receptors to hormones and the selective permeability of biological membranes, regulate the expression of many genes, have an antioxidant effect, and activate energy enzymes exchange, which ultimately leads to the economization of the metabolism and adaptation of the body to the unfavorable environment [3]. Due to their unique properties, many natural adaptogens are widely used, such as ginseng (*Panax ginseng* C.A.Mey.), roseroots (*Rhodiola rosea* L., *Sedum roseum* (L.) Scop.), and devil’s bush (*Eleutherococcus senticosus* (Rupr. & Maxim.) Maxim.) [4], while other adaptogens are currently being comprehensively researched and implemented in pharmacological practice.

**Figure 1:**
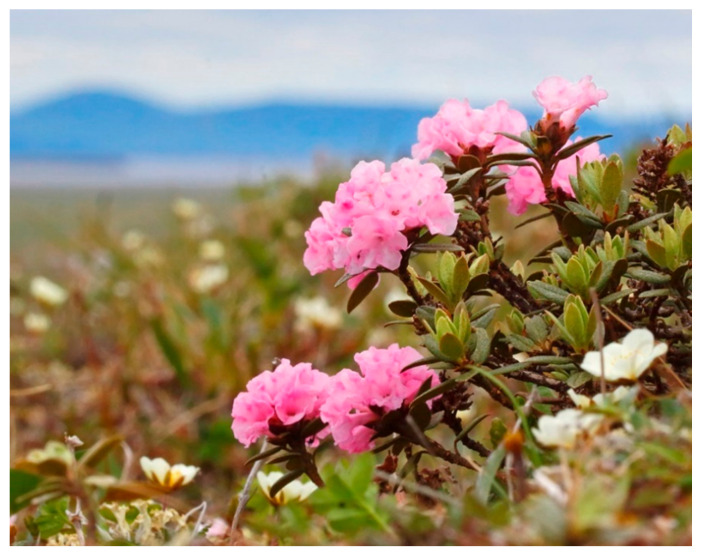
*Rhododendron adamsii* Rehder (Sagan Dalya) in its natural habitat (Republic Sakha Yakutia, Lena River delta, Kubalakh-Aryta Island, Orto-Khaya Mountain, tundra).

Globally, *Rhododendron adamsii* Rehder {*R. fragrans* (Adams) Maxim., *Azalea fragrans* Adams}, or Adams’ rhododendron (Figure 1), is a lesser-known adaptogen species, however, it is widely used in Siberia. *Rhododendron adamsii* is a small, evergreen shrub of the Ericaceae family, with dense rusty branches and thick, leathery, matt green wintering leaves [5]; when the leaves of the plant are touched, a greasy aromatic wax that thickly covers the leaves remains on the skin. *Rhododendron adamsii* grows in the mountains in the subalpine zone and, less commonly, in the alpine and upper forest zones of Central and Eastern Siberia and the Far East. 

*Rhododendron adamsii* has numerous folk names, such as Sagan Dalya tea, White Wing, or Belgorod tea, owing to the story that the mountain spirit lives in it and helps to recover the health of warriors. The history of the Sagan Dalya name is enveloped in many poetic legends; according to one such legend, two lovers, Sagan and Dalya were separated by the evil shamaness, causing tears of the girl to fall to the ground and turn into the evergreen flowering shrubs also known as White Wing or Sagan Dalya [6]. In Buddhist mythology, Sagan Dalya is one of the seven plants surrounding the teacher of healing, the All-Enlightened Bhaishajyaguru [7].

Ethnopharmacological data indicates that the Buryat medicinal decoction of *R. adamsii* leaves (*саган дали*) is used as an elixir to strengthen the human organism [8]. Yakutian nomads used *R. adamsii* (*хаскара*) decoction as a stimulant, diaphoretic, antipyretic, antibacterial, and analgesic drug [9]. In Mongolia, Buryatia, and Altai, shamans traditionally drink *R. adamsii* decoction as a tonic beverage, to enter a trance, and as a panacea for any disease. In Tibet, *R. adamsii* decoction is used to treat nervous disorders while, throughout Siberia, it is known as a powerful energy drink [10].

Current scientific information concerning *R. adamsii* metabolites remains insufficient, despite public interest and the wide use of this plant. Siberian samples of *R. adamsii* leaves and stems have been studied via GC/MS to elucidate their essential oil compositions, with nerolidol (9–29%), β-farnesene (9–35%), 4-phenyl-2-butanone (3–12%), and aromadendrene (3–10%) found to be the dominant compounds [11]. Some flavonoids have been characterized in ethanol extracts of R. adamsii leaves and stems, including myricetin, quercetin, dihydroquercetin, and rutin [12]. A recent study of the CO_2_ extract of *R. adamsii* leaves and branches showed a predomination of lipophilic components, including fatty acids, sterols, triterpenes, and some phenolic aglycones (flavonoids, coumarins) [13]. Additionally, at present, no data exists regarding the methods of application and the biological activity of *R. adamsii*, which has led to the emergence of many legends, as well as outright speculation, concerning the effectiveness of this plant.

As part of the ongoing work involving the metabolomic study of Siberian rhododendrons [14,15,16], we detail the first analysis of methanolic extracts of *R. adamsii* extracts in relation to seasonal metabolite variation using HPLC-PDA-ESI-tQ-MS (high-performance liquid chromatography with photodiode array detection and electrospray ionization triple quadrupole mass spectrometric detection) techniques. For the first time, the antioxidant and adaptogenic potentials of *R. adamsii* extracts were studied, demonstrating high effectiveness.

## 2. Materials and Methods

### 2.1. Plant Material and Chemicals

All samples of *Rhododendron adamsii* (215 samples totally) were collected in the tundra habitat in Republic Sakha Yakutia, Lena River delta, Kubalakh-Aryta Island, Orto-Khaya Mountain (72°26′22.0″ N, 126°18′09.0″ E, 280 m a.s.l.) in January (15.I.2019–17.I.2019; 15 samples), March (12.III.2019–15.III.2019; 19 samples), May (16.V.2019–20.V.2019; 26 samples), June (12.VI.2019–16.VI.2019; 31 samples), July (15.VII.2019–17.VII.2019; 42 samples), August (17.VIII.2019–22.VIII.2019; 36 samples), October (12.X.2019–15.X.2019; 27 samples), and December (19.XII.2019–23.XII.2019; 19 samples). One sample consisted of 10–20 leaves (average length 2 cm, height 1 cm) collected from one bush. The species was authenticated by Dr. N.I. Kashchenko (IGEB SB RAS, Ulan-Ude, Russia). The plant material was dried in the ventilated heat oven at 40 °C within 7–10 days and stored at 3–4 °C before analysis. The reference compounds were purchased from Cayman Chemicals (Ann Arbor, MI, USA), ChemFaces (Wuhan, Hubei, China), Extrasynthese (Lyon, France), MCE Med Chem Express (Monmouth, NJ, USA), Sigma-Aldrich (St. Louis, MO, USA), and Wuhan Chem Norm Biotech Co., Ltd. (Wuhan, China) (Table S1).

### 2.2. Plant Extracts Preparation

The extracts of *R. adamsii* leaves for the general chemical composition and preliminary bioactivity study were prepared from the powdered plant samples (50–100 g) extracted by appropriate solvent (water, methanol 20–100%) with sonication (40 min, 40 °C, ultrasound power 100 W, frequency 35 kHz). The chilled (20 °C) liquid was consequently filtered (filter paper), concentrated in vacuo until dryness, milled, and stored at 4 °C before analysis. The seasonal variation of metabolites and bioactivity of *R. adamsii* leaves analyzed for the extracts obtained by using 40% methanol as a solvent in the same extraction conditions.

### 2.3. Chemical Composition Analysis

UV-Vis spectrophotometer SF-200 (OKB Spectr, Saint Petersburg, Russia) was used for spectrophotometric quantitative determination of total protein (as mg/g BSA equivalents) [17], total soluble carbohydrates (as mg/g glucose equivalents) [18], polysaccharides (as mg/g glucose equivalents) [19], phenolics (as mg/g gallic acid equivalents) [20], flavonols (as mg/g hyperoside equivalents) [21], flavanols (as mg/g taxifolin equivalents) [22], catechins (as mg/g (+)-catechin equivalents) [23], and procyanidins (as mg/g procyanidin B_1_ equivalents) [24] in *R. adamsii* extracts. All the analyses were carried out five times and the data were expressed as mean value ± standard deviation (S.D.).

### 2.4. Antioxidant Activity

Microplate spectrophotometric assays were used to study the scavenging activity of *R. adamsii* extracts against 2,2-diphenyl-1-picrylhydrazyl radicals (DPPH^•^) [25], 2,2′-azino-bis(3-ethylbenzothiazoline-6-sulfonic acid) cation radicals (ABTS^•^^+^) [26], *N*,*N*-dimethyl-*p*-phenylenediamine radicals (DMPD^•+^) [27], superoxide anion radicals (O_2_^•^^−^) [28], hydroxyl radicals (^•^OH) [28], and chloride radicals (Cl^•^) [29]. Carotene bleaching spectrophotometric assay used β-carotene as a substrate (Sigma-Aldrich, cat. No. C9750) [30] and nitric oxide (II) scavenging assay used sodium nitroprusside as NO source [31]. Ferrous (II) ion chelating activity was studied by spectrophotometric assay [32]. Trolox (cat. No. 238813, ≥97%; Sigma-Aldrich) was used as a reference standard (1–100 μg/mL in methanol). All the analyses were carried out five times and the data were expressed as mean value ± standard deviation (S.D.).

### 2.5. Adaptogenic Activity

#### 2.5.1. One-Step Swimming to Exhaustion Test

The mice (*n* = 90) were randomly divided into ten groups received saline (0.9% NaCl; 0.5 mL), *R. adamsii* leave extract obtained by the various solvent (0–100% methanol) at dose 50 mg/kg, and *Rodiola rosea* rhizome extract (5% rosavins, Vitaforest Ltd., Saint Petersburg, Russia; 50 mg/kg). In 60 min, the mice were individually placed in a glass cylinder (height 40 cm, diameter 20 cm) filled with water (height 15 cm; 22 ± 1 °C) and exhaustive swimming of rodents continued until the first immersion in the water. After the swimming sessions, the mice were towel-dried and returned to their housing. Each animal was used only once. The swimming time was measured using a stopwatch and was expressed in min. The experiment was realized in two versions, the first was the one-day application of plant extracts and the second was the 10-day application of plant extracts.

#### 2.5.2. Two-Step Swimming to Exhaustion Test

The mice (*n* = 54) were randomly divided into six groups received saline (0.9% NaCl; 0.5 mL), *R. adamsii* leave extract (January, May, July, October samples) at dose 50 mg/kg, and *R. rosea* extract (50 mg/kg) during 10 days and on the 10th day, the rodents were tested as described in Section 2.5.1. One hour later, the mice have been retested in the same conditions. The swimming times at each step were measured using a stopwatch and were expressed in min. At the end of the experiment, laboratory animals were decapitated and the homogenates of skeletal muscles (quadriceps femoris) and liver, and blood serum were assayed for the following biochemical parameters: skeletal muscles—adenosine triphosphate (fluorimetric ATP assay kit; Sigma-Aldrich, cat. No. MAK190), creatine phosphate (colorimetric phosphocreatine PCr ELISA kit; Abbexa Ltd., Cambridge, UK, cat. No. abx258965) and lactate (fluorimetric lactate assay kit; Sigma-Aldrich, cat. No. MAK064); blood serum—pyruvic acid (fluorimetric pyruvate assay kit; Sigma-Aldrich, cat. No. MAK071), glucose (colorimetric glucose assay kit; Sigma-Aldrich, cat. No. MAK264), malondialdehyde (colorimetric MDA assay kit; Abcam, Cambridge, UK, cat. No. ab238537), and catalase (catalase colorimetric activity kit; Invitrogen, Carlsbad, CA, USA, cat. No. EIACATC); liver—glycogen (colorimetric glycogen assay kit; Cayman Chemical, Ann Arbor, MI, USA, cat. No. 700480).

### 2.6. Polyamide Solid-Phase Extraction (SPE)

The pre-chromatographic solid-phase extraction (SPE) of *R. adamsii* extract was used before HPLC separation as described before [33,34] with a slight modification. Polyamide cartridges Chromabond (Polyamide 6; 6 mL, 1000 mg; Sorbent Technologies, Inc., Norcross, GA, USA) were eluted with methanol (70 mL) and water (90 mL). The extract of *R. adamsii* (100 mg) was dissolved in 90% methanol (1 mL) followed by dilution of 20% methanol (8 mL), then centrifuged (6000× *g*, 15 min), and the supernatant was transferred in the volumetric flask (10 mL). Aliquots of internal standards were added to volumetric flask including 20-hydroxyecdysone (100 μL; 500 μg/mL in 50% methanol; internal standard-I), apigenin-7-*O*-glucoside (150 μL; 400 μg/mL in 70% methanol; internal standard-II), apigenin-7-*O*-glucuronide (150 μL; 400 μg/mL in 70% methanol; internal standard-III), and epigallocatechin 3-*O*-gallate (100 μL; 1000 μg/mL in 30% methanol; internal standard-IV). The final volume reached 10 mL with 25% methanol in the volumetric flask (solution A). Solution A (2 mL) was passed through polyamide SPE-cartridge and eluted with water (40 mL; eluate I), methanol (50 mL; eluate II), 0.55% NH_3_ in methanol (50 mL; eluate III), pure water (120 mL), and DMSO (40 mL; eluate IV). Eluates I–IV stored at 4 °C before chromatographic analysis (Section 2.7).

### 2.7. High-Performance Liquid Chromatography with Photodiode Array Detection and Electrospray Ionization Triple Quadrupole Mass Spectrometric Detection (HPLC-PDA-ESI-tQ-MS)

Qualitative chromatographic analysis of metabolic profiles of *G. bifida* extracts was done by high-performance liquid chromatography with photodiode array detection and electrospray ionization triple quadrupole mass spectrometric detection (HPLC-PDA-ESI-tQ-MS) technique using a liquid chromatograph LC-20 Prominence coupled with photodiode array detector SPD-M30A (wavelength range 200–600 nm), and triple-quadrupole mass spectrometer LCMS 8050 (all Shimadzu, Columbia, MD, USA) and C18 columns. Two-eluent gradient elution was used for the successful separation of compounds in four chromatographic modes (HPLC conditions) described in Table 1. 

The injection volume was 1 μL in all modes. The UV-Vis spectra were registered in the spectral range of 200–600 nm. Mass spectrometric detection was performed in negative ESI mode and the temperature levels of ESI interface, desolvation line, and heat block were 300 °C, 250 °C, and 400 °C, respectively, and the flow of nebulizing gas (N_2_), heating gas (air), and collision-induced dissociation gas (Ar) were 3 L/min, 10 L/min, and 0.3 mL/min, respectively. The mass spectra were registered as 3 kV source voltage and collision energy −10–35 eV by the scanning range of *m*/*z* 80–2000. The managing of the LC-MS system was realized by LabSolution’s workstation software equipped with the inner LC-MS library. The final identification of metabolites performed after an integrated analysis of retention time, ultraviolet, and mass spectra in comparison with the reference standards and literature data.

### 2.8. Metabolite Quantification

The quantitative analysis of compounds found in *R. adamsii* was done using the above described HPLC-PDA-ESI-tQ-MS conditions (Section 2.7) and full scan MS peak area used for calculation. One hundred and one compounds were quantitatively analyzed using 55 reference standards (Table S1). Each compound was carefully weighed (10 mg), dissolved in the methanol-DMSO mixture (1:1) in volumetric flasks (10 mL), and the reference standard calibration curves were built using the stock solutions in methanol (1–100 µg/mL). Mass spectrometric peak area data were used to plot ‘concentration–peak area’ graphs and determination the validation criteria (correlation coefficients, *r*^2^; standard deviation, *S*_YX_; limits of detection, LOD; limits of quantification, LOQ; and linear ranges) calculated as described previously [35] (Table S2). All quantitative analyses were carried out five times, and the data were expressed as mean value ± standard deviation (S.D.). 

### 2.9. HPLC-UV Assay Coupled with DPPH Precolumn Incubation

The extract of *R. adamsii* (July sample, 100 mg) was sonically dissolved in 40% methanol (5 mL), centrifuged (6000× *g*, 20 min), and used for HPLC-PAD separation without SPE pretreatment. The aliquote of *R. adamsii* extract solution (50 μL) was mixed with 0.5% DPPH^•^ radicals solution in methanol (50 μL) and incubated 15 min at 20 °C. The probe without DPPH^•^ radicals preincubation was diluted with methanol (1:1) before separation. High-performance liquid chromatography with UV detection coupled with DPPH^•^ radicals preincubation performed by microcolumn liquid chromatography MiliChrom A-02 (EcoNova, Novosibirsk, Russia). The apparatus was coupled with UV detector 190-360 EcoNova (EcoNova, Novosibirsk, Russia) and ProntoSIL-120-5-C18 AQ column (50 × 1 mm, 1 μm; Metrohm AG, Herisau, Switzerland) with column temperature of 30 °C. The eluent composition was 0.2 M LiClO_4_ in 0.01 M HClO_4_ for eluent A and 0.01 M HClO_4_ in MeCN for eluent B. The injection volume was 1 μL, and the elution flow was 150 μL/min. The gradient separation was used programmed as 0.0–26.6 min 5–100% B, 26.6–28.6 min 100% B. The chromatograms were recorded at 270 nm. Finally, the chromatograms of untreated and DPPH^•^ radicals probes were overlapped and compared. The reduction of chromatographic peak area indicating the radical-scavenging potential of compounds eluted in corresponding peaks.

### 2.10. Statistical and Multivariate Analysis

Statistical analyses were performed by one-way analysis of variance, and the significance of the mean difference was determined by Duncan’s multiple range test. Differences at *p* < 0.05 were considered statistically significant. The results are presented as mean values ± standard deviations (S.D.) of some replicates. The linear regression analysis and generation of calibration graphs were conducted using Advanced Grapher 2.2 (Alentum Software Inc., Ramat-Gan, Israel). Principal component analysis based on a data matrix (171 markers × 215 samples) was performed using Graphs 2.0 utility for Microsoft Excel (Komi NTc URO RAN, Syktyvkar, Russia) to generate an overview for group clustering.

## 3. Results and Discussion

### 3.1. Chemical Composition and Bioactivity of Rhododendron Adamsii Leaves: Impact of Solvent Type 

Prior to the in-depth chemical and biological study of *R. adamsii* leaves, we analyzed various extracts to identify the best solvent for providing the most active remedy. Various methanols (0–100%) were used and characterized chemically (Table 2). The total yield of the extracts varied from 12.5% to 35.0% of dry plant weight. The basic nutrient content of *R. adamsii* leaves fluctuated depending on the methanol concentration: proteins and soluble carbohydrates showed the highest amounts in water extractions (23.69 mg/g and 326.03 mg/g, respectively), while lipids dominated in the pure methanol extract (254.12 mg/g). The main explanation for the distribution of nutrients in extracts lies in the rule ’like solves like’, i.e., hydrophilic solvents extract hydrophilic compounds, and vice versa; the same rule applies to polysaccharides due to their high content in the water extractions (57.60 mg/g). 

The phenolics are a class of compounds with a medium polarity that demonstrate optimal extractability using 40% methanol, with yields of 293.35 mg/g for total phenolic content, 138.88 mg/g for total flavonols, 16.84 mg/g for flavanols, 89.27 mg/g for catechins, and 27.98 mg/g for procyanidins. This impacted the radical scavenging ability of *R. adamsii* extracts against 2,2-diphenyl-1-picrylhydrazyl radicals (DPPH**^•^**), well-known artificial free radicals used in the detection of plant antioxidants [36]. The 40% methanol extract demonstrated the best DPPH scavenging activity with IC_50_ value 4.82 μg/mL (half maximal inhibitory concentration). The use of solvents with lower or higher concentrations of methanol negatively affects the antioxidant potential of the extracts.

Early studies of rhododendrons showed good ability to accumulate phenolic compounds such as *R. ponticum* (349.53 mg/g) [37], *R. pseudochrysanthum* (319 mg/g), *R. breviperulatum* (265 mg/g), *R. oldhamii* (264 mg/g) [38], *R. anthopogonoides* (165 mg/g) [39], *R. jasminiflorum* (48.11 mg/g), *R. konori* (41.17 mg/g), and *R. javanicum* (40.39 mg/g) [40]. Flavonoids seem to be the major compound in *R. ponticum* (311.16 mg/g) [37], *R. anthopogonoides* (231.37 mg/g) [39], *R. jasminiflorum* (8.92 mg/g), *R. javanicum* (6.15 mg/g), and *R. seranicum* (5.90 mg/g) [40]. Information regarding total contents of the separated phenolic classes, such as flavonols, flavanols, catechins, and procyanidins, is not widely available from rhododendrons around the world. Despite this, the high antioxidant potential of rhododendrons is a well-known fact, as shown by the DPPH^•^ values of *R. ponticum* (IC_50_ 1.23 μg/mL) [37], *R. pseudochrysanthum*, *R. oldhamii* (IC_50_ 7.5 μg/mL), *R. kanehirai* (IC_50_ 7.7 μg/mL) [38], *R. przewalskii* (IC_50_ 31 μg/mL) [41], *R. anthopogonoides* (IC_50_ 63.75 μg/mL) [39], *R. jasminiflorum* (29 μM trolox/g), *R. seranicum* (28 μM trolox/g), and *R. javanicum* (26 μM trolox/g) [40] extracts. 

**Figure 2:**
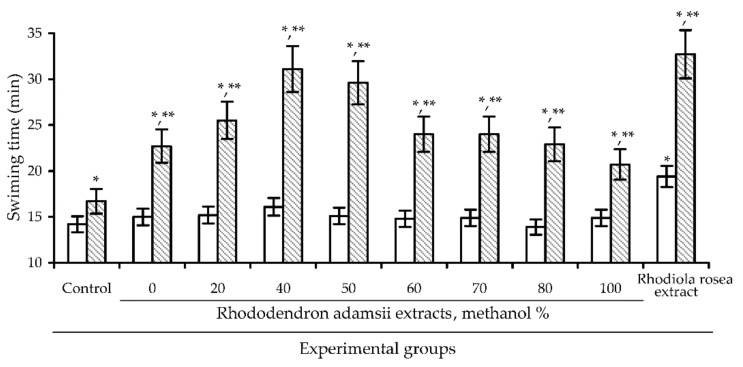
The effect of *R. adamsii* leaf extracts (solvent: 0–100% methanol) and *R. rosea* extract (50 mg/kg) on swimming time of mice in swimming to exhaustion test on day 1 (empty bars) and day 10 (shaded bars). *—*p* < 0.05 vs. control group, day 1; **—*p* < 0.05 vs. control group, day 10.

Unlike the antioxidant properties of *Rhododendron* species, the impact of rhododendron extracts on physical endurance has not been studied previously, therefore, the adaptogenic potential of *R. adamsii* is disputed and requires further investigation; in our current study, we use the one-step swimming to exhaustion test of mice to examine this, using swimming time as an indicator of effectiveness. The extract of *R. rosea* roots (total rosavins 5%) was used as a plant remedy known to enhance resistance activity to physical training [28]. The eight extracts of *R. adamsii* leaves created using 0–100% methanol as a solvent were studied using a single dose of 50 mg/kg, the same dose as used for *R. rosea* extract (Figure 2). Preliminary experiments demonstrated lower (25 mg/kg) and higher doses (100 mg/kg) of the extracts to be less effective.

**Figure 3:**
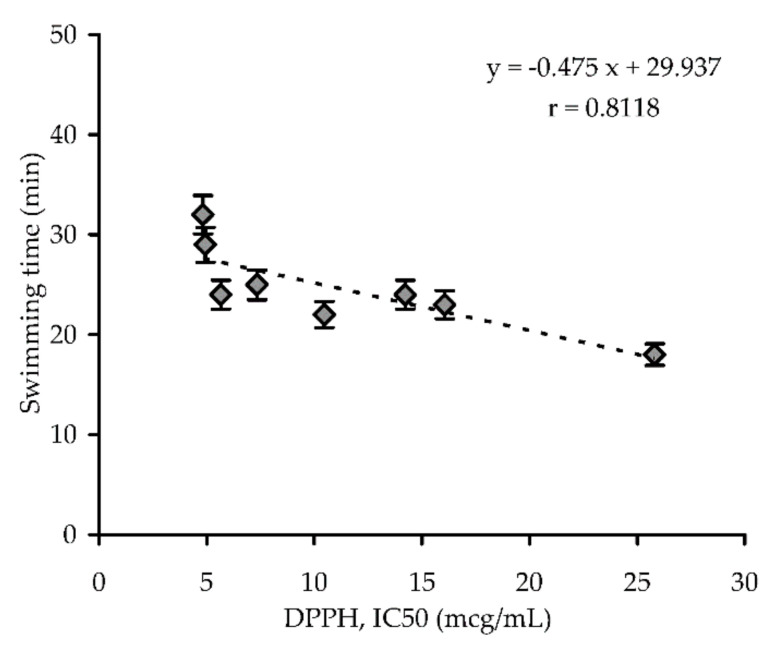
Correlation graph between radical scavenging ability of *Rhododendron adamsii* extracts against 2,2-diphenyl-1-picrylhydrazyl radicals (DPPH; independent variable *x* in regression equation) and swimming time of mice in swimming to exhaustion test on day 10 (dependent variable *y* in regression equation). r—correlation coefficient.

In the swimming test of mice experiments, one-day application of *R. adamsii* extract resulted in no statistically significant (*p* < 0.05) increase of swimming time of mice, in contrast to *R. rosea* extract, which resulted in a 36% increase in swimming time compared with the control group (19.4 min vs. 14.2 min, respectively). The ten-day experiments demonstrated increases in animal endurance in all *R. adamsii* extract groups, with various levels of intensity. Of the extract groups, the 40% methanol extract showed the best effectiveness (31.1 min) when compared to the *R. rosea* extract group (32.7 min). Based on these results, we can conclude that *R. adamsii* extract enhances physical endurance, however, the properties of this endowment differ from those provided by *R. rosea*, as the latter medicine is characterized by a rapid influence on the animal subject while the *R. adamsii* extract’s impact develops over time (cumulative effect). It is easily noted that the 40% methanol extract of *R. adamsii* leaves demonstrated the maximal antioxidant power and adaptogenic potential, indicating a possible linkage of both activities. Interestingly, a strong correlation between the radical scavenging ability of *R. adamsii* extracts against DPPH radicals and the swimming time of mice in swimming to exhaustion test on day 10 was observed with correlation coefficient r = 0.8118 (Figure 3). Some researchers have previously expressed their views on the role of antioxidants in the realization of adaptogenic properties of plant extracts; these researchers have a cautious view that antioxidants decrease the risk of complications induced by oxidative stress, thus contributing to the positive effect of adaptogens [42,43]. In light of known data concerning stress-induced increases in free radical processes in mammals [1], it makes sense that a close relationship between adaptogens and antioxidant properties exists. 

### 3.2. Rhododendron Adamsii Leaves Metabolites: LC-MS Characterisation and Seasonal Variation

*Rhododendron* genus demonstrates the presence of numerous chemical classes with specific chromatographic behavior [9]. Critical to the success of metabolite separation are the preliminary procedures for purification and partitioning of the total plant extract on the specific fractions. Solid-phase extraction (SPE) is a helpful tool for the pre-treatment of plant samples prior to chromatographic analysis [28,33,34]. Application of polyamide-based SPE of methanol extract of *R. adamsii* leaves yielded four fractions enriched with highly hydrophilic compounds (SPE-1), neutral phenolics (SPE-2), acidic and acylated phenolics (SPE-3), and tannin-like compounds (SPE-4); this method avoided overlapping of the chromatographic zones and enabled better identification of the compounds.

**Figure 4:**
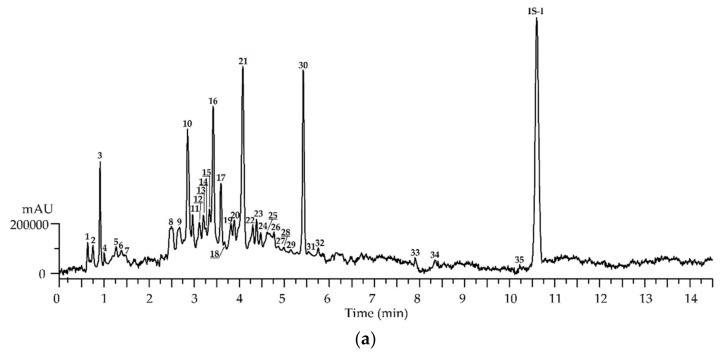

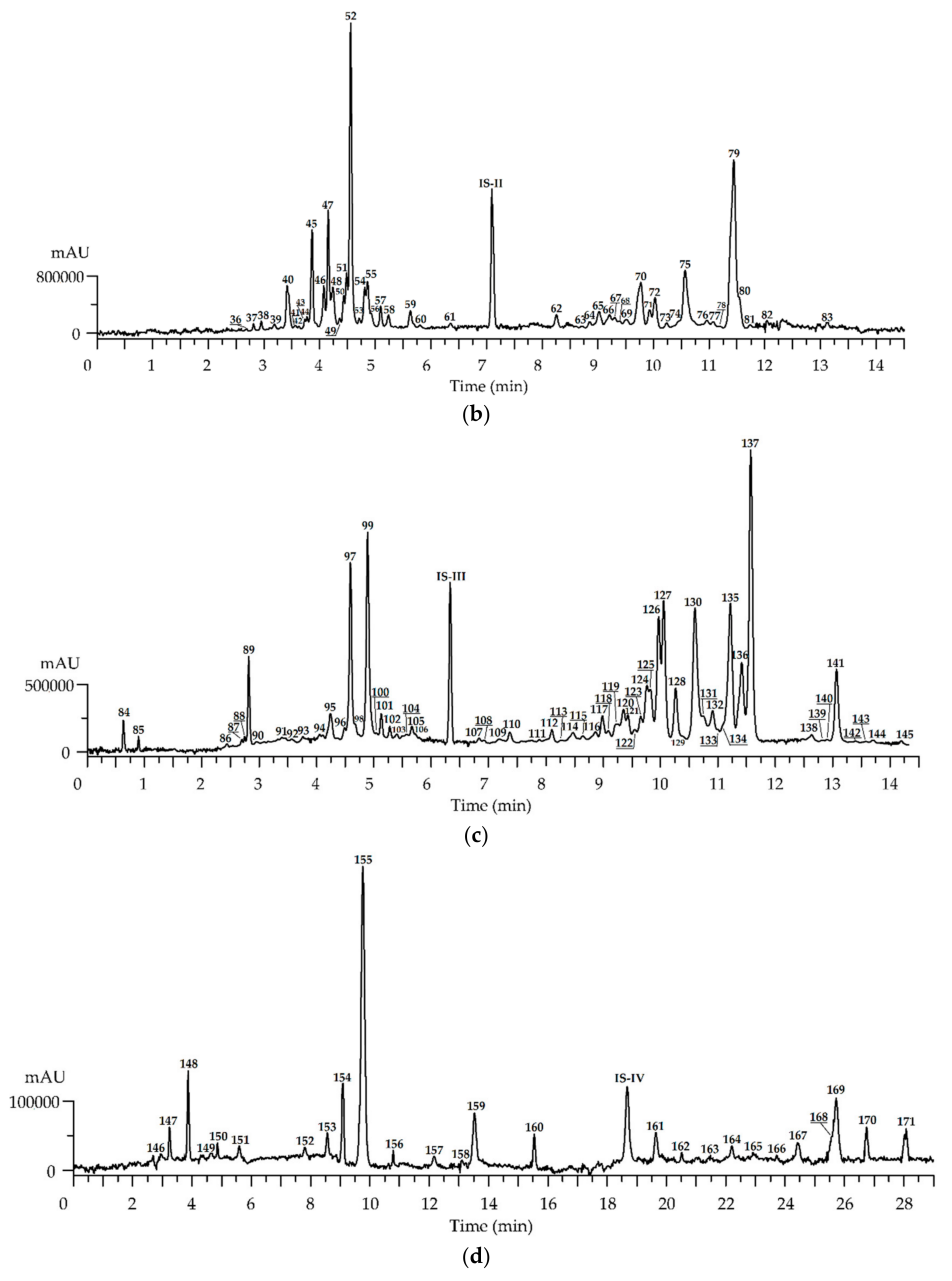
High-Performance Liquid Chromatography with Electrospray Ionization Triple Quadrupole Mass Spectrometric Detection (HPLC-ESI-tQ-MS) chromatogram (Total Ion Chromatogram (TIC) mode, negative ionization) of solid-phase extraction (SPE) eluates of *R. adamsii* leaves (July sample) extract: H_2_O eluate (SPE-1; **a**), MeOH eluate (SPE-2; **b**), NH_3_-MeOH eluate (SPE-3; **c**), DMSO eluate (SPE-4; **d**). Compounds are numbered as listed in Table 2. Internal standards used: 20-hydroxyecdysone (IS-I), apigenin-7-*O*-glucoside (IS-II), apigenin-7-*O*-glucuronide (IS-III), epigallocatechin *O*-gallate (IS-IV).

**Figure 5:**
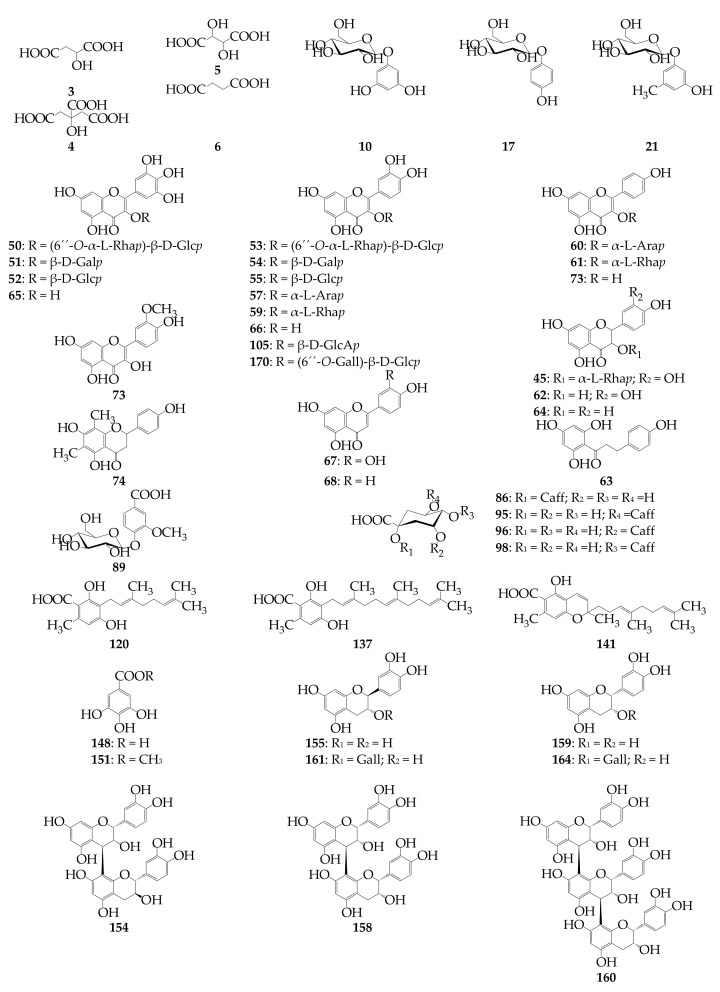
Structures of known compounds found in *R. adamsii*. Abbreviation used: Ara*p*—arabinopyranose; Caff—Caffeoyl; Gall—Galloyl; Gal*p*—Galactopyranose; Glc*p*—Glucopyranose; GlcA*p*—Glucuronopyranose; Rha*p*—Rhamnopyranose.

To separate the majority of the methanol-extractable compounds of the *R. adamsii* leaves, we used high-performance liquid chromatography with photodiode array and electrospray ionization triple quadrupole mass spectrometric detection (HPLC-PDA-ESI-tQ-MS). Synchronous analysis of chromatographic mobility, ultraviolet spectra, and mass spectral pattern, and comparison of the data with reference standards or/and literature information [12,13,26,28,28,33,35,44,45,46,47,48,49,50,51,52,53,54,55] resulted in the identification of 171 compounds (Figure 4 and Figure 5, Table 3 and Table S3). Previously, only ten phenolics had been described in *R. adamsii* leaves, resulting in more than 160 originally documented here [11,12,13].

#### 3.2.1. Carbohydrates and Organic Acids

The highly hydrophilic compounds of *R. adamsii* leaves comprised two carbohydrates—*O*-hexosyl-hexose (**1**) and hexose (**2**), and five organic acids—malic (**3**), citric (**4**), tartaric (**5**), succinic (**6**), and fumaric acids (**7**). The nature of the carbohydrates was studied using high-performance anion-exchange chromatography [56], which showed the presence of saccharose, raffinose, glucose, fructose, and galactose (Figure S1).

#### 3.2.2. Simple Phenol Glycosides

Fifteen phenolic glycosides were found in *R. adamsii*, mostly derivatives of phloroglucinol (**8**–**14**), hydroquinone (**15**–**17**, **19**, **20**), and orcinol (**18**, **21**, **24**). Phloroglucinols were in the form of di-*O*-hexosides (**8**, **9**), *O*-glucoside (phlorin, **10**), di-*O*-hexoside-*O*-acetates (**11**, **12**), and di-*O*-hexoside-di-*O*-acetates (**13**, **14**). Previous data regarding phloroglucinol presence in the *Rhododendron* genus includes 1-*O*-acetylphloroglucinol found in *R. ferrugineum* [44], therefore, phloroglucinol glycosides were detected here for the first time in the genus. The hydroquinone glycoside, arbutin (**17**), typical for some Ericaceae plants [45], was identified in *R. adamsii* using a reference standard; this compound had previously been detected in *R. latoucheae* [57] and *R. arboreum* [58]. Some rare hydroquinone glycosides were discovered, such as hydroquinone di-*O*-hexosides (**15**, **16**) with the possible structure of known 6-*O*-glucosyl arbutin [45], an unknown hydroquinone di-*O*-hexoside-*O*-methyl ester (**19**), and a hydroquinone *O*-hexoside-*O*-methyl ester (**20**) that is probably methylarbutin [46]. 

Sakakin (**21**), the orcinol *O*-glucoside that was identified after comparison with the reference standard, was found in rhododendrons for the first time, although orcinol itself has previously been detected in *R. dauricum* twigs [47]. Additionally, orcinol di-*O*-hexoside (**18**) and its *O*-acetate (**24**) were components of *R. adamsii* leaves. The unusual combination of simple phenolic glycosides in *R. adamsii* probably relates to its specific metabolomic features, which are extraordinary for the *Rhododendron* genus but understandable for the Ericaceae family.

#### 3.2.3. Triterpene Glycosides

Preliminary analysis of SPE-1 eluate using acidic hydrolysis followed by HPLC separation allowed the detection of ursolic acid and unquantifiable traces of oleanolic acid (Figure S2). The use of the LC-MS technique showed thirteen ursolic acid derivatives as tri-*O*-hexosides (**22**, **23**), di-*O*-hexosides (**25**, **26**), tri-*O*-hexoside-*O*-acetates (**27**, **28**), di-*O*-hexoside-*O*-acetate (**29**), *O*-hexoside (**30**), di-*O*-hexoside-di-*O*-acetates (**31**, **32**), *O*-hexoside-*O*-acetates (**33**, **34**), and *O*-hexoside-di-*O*-acetate (**35**) in the *R. adamsii* leaves. The results of earlier studies of *R. adamsii* [59,60] demonstrated the presence of a low level of free ursolic acid (<1 mg/g), owing to the domination of the glycosylated form of triterpene acids in the plant.

#### 3.2.4. Flavonoids

Sixty-eight compounds found in various SPE eluates of *R. adamsii* leaves extract were determined as flavonoids and separated into flavonols (52 compounds), dihydroflavonols (13 compounds), and flavones (3 compounds) in glycoside and aglycone states. 

Flavonols belonged to one of three groups depending on their aglycone structure—myricetin, quercetin, or kaempferol. The myricetin group was the largest, with twenty-seven members including neutral, acidic, and acylated derivatives. After comparison with reference standards, some neutral myricetins were identified as myricetin-3-*O*-rutinoside (**50**), myricetin-3-*O*-galactoside (**51**), myricetin-3-*O*-glucoside (isomyricitrin, **52**), and myricetin-3-*O*-rhamnoside (myricitrin, **58**). The remaining compounds observed in mass spectra the loss of pentose fragments with *m*/*z* 132 (myricetin *O*-pentoside, **56**) or fragments of hexose (*m*/*z* 162) and desoxyhexose (*m*/*z* 146) in ratios of 1:1 (myricetin *O*-hexoside-*O*-desoxyhexoside, **49**), 2:1 (myricetin di-*O*-hexoside-*O*-desoxyhexoside, **47**), 2:2 (myricetin di-*O*-hexoside-di-*O*-desoxyhexoside, **46**), 3:2 (myricetin tri-*O*-hexoside-di-*O*-desoxyhexoside, **37**), or 3:3 (myricetin tri-*O*-hexoside-tri-*O*-desoxyhexoside, **36**). 

The known analogs of **56** are myricetin-3-*O*-arabinoside and myricetin-3-*O*-xyloside from *R. anthopogonoides* [49]. Isomeric to **50**, flavonol **49** is most likely myricetin-3-*O*-neohesperidoside found in *Physalis angulata* [50], while **47** is close to myricetin-3-*O*-rutinoside-7-*O*-glucoside from *Limnanthes douglasii* [51]. The myricetins with hexose/desoxyhexose ratio 2:2–3:3 remain unknown.

Acidic myricetin glycosides have fragments of hexuronic acid and hexose in ratios of 1:0 (myricetin *O*-hexuronide, **99**), 1:1 (myricetin *O*-hexoside-*O*-hexuronide, **97**), 1:2 (myricetin *O*-hexoside-di-*O*-hexuronide, **94**), 2:1 (myricetin di-*O*-hexoside-*O*-hexuronide, **93**), or 2:2 (myricetin di-*O*-hexoside-di-*O*-hexuronide; **91**, **92**). We were able to find information detailing one myricetin-3-*O*-glucuronide (the analog of **99**) described in many plant families, among them the ericaceous genus *Richea* [61] although none in *Rhododendron*. Acylated myricetins contained both neutral and acidic carbohydrates, such as *O*-hexoside-*O*-acetates (**107**,**111**,**118**,**119**), *O*-hexoside-*O*-gallates (**165**–**169**), and *O*-hexouronide-*O*-acetates (**106**,**108**), while none of them have analogs or close structure among the known phytochemicals.

The identified quercetin glycosides were rutin (**53**), hyperoside (**54**), isoquercitrin (**55**), avicularin (**57**), quercitrin (**59**), miquelianin (**15**), and quercetin-3-*O*-(6´´-*O*-galloyl)-glucoside (**170**). Compounds **53**, **57**, and **59** are known flavonoids of *R. adamsii* [12,13], while **54** and **55** have been reported in many rhododendrons [49]. The unknown non-acylated quercetins were *O*-hexoside-*O*-desoxyhexosides (**38**, **48**) and *O*-hexoside-*O*-hexuronides (**100**, **101**, **103**, **104**), while acyl-fragments gave *O*-hexoside-*O*-acetates (**121**, **122**), *O*-hexuronide-*O*-acetates (**110**, **114**, **115**), and *O*-hexoside-*O*-gallate (**171**). Among kaempferols, we detected only two derivatives, juglanin (**60**) and afzelin (**61**), both previously described in rhododendrons [49]. Free myricetin (**65**), quercetin (**66**), and kaempferol (**73**) have previously been isolated from *R. adamsii* leaves [12,13], while isorhamnetin (**69**) was found for the first time here. 

Dihydroflavonols found in *R. adamsii* were derivatives of dihydromyricetin (ampelopsin), dihydroquercetin (taxifolin), and dihydrokaempferol (aromadendrin). Dihydromyricetin detected in leaves of *R. decorum* and *R. mucronulatum* [49] was found as two glycosides, di-*O*-hexoside (**39**) and *O*-hexoside (**40**), both with no analogs in plants. Only dihydromyricetin *O*-pentoside has been previously characterized as a component of *R. ferrugineum* [51]. Free dihydroquercetin (taxifolin, **62**) is a known flavonoid of *R. adamsii* [12], unlike nine of its glycosides, which were *O*-hexoside (**44**), 3-*O*-rhamnoside (astilbin, **45**), *O*-hexuronide (**102**), n-*O*-hexoside-n-*O*-desoxyhexosides (**41**–**43**), and *O*-hexuronide-n-*O*-acetates (**109**, **112**, **113**). Taxifolin glycosides have been isolated from various rhododendrons, including 3-*O*-xyloside and 3-*O*-rhamnoside from *R. spinuliferum* and 3-*O*-arabinoside from *R. mucronulatum* and *R. ferrugineum* [49]. Hexosides and desoxyhexosyl-hexosides of dihydroquercetin are newly demonstrated in the *Rhododendron* genus. Free dihydrokaempferol (aromadendrin, **64**) has previously been described in *R. decorum* [49], although this is the first time it has been identified in *R. adamsii*. 

The trace flavonoid group of *R. adamsii* included flavones identified as luteolin (**67**), apigenin (**68**), and farrerol (**74**) after comparison with reference standards. Farrerol has previously been described in *R. adamsii* [13] and *R. dauricum* [49].

#### 3.2.5. Prenylated Phenols

A rare group of *Rhododendron* metabolites, prenylated phenols or phytocannabinoids, that were found in *R. adamsii* include three structural type-like derivatives of cannabigerorcinic acid (**70**–**72**, **75**–**83**, **116**, **117**, **120**, **123**, **124**, **129**), grifolic acid (**125**–**128**, **130**–**132**, **137**–**140**), and daurichromenic acid (**133**–**136**, **141**–**145**) [62]. 

**Figure 6:**
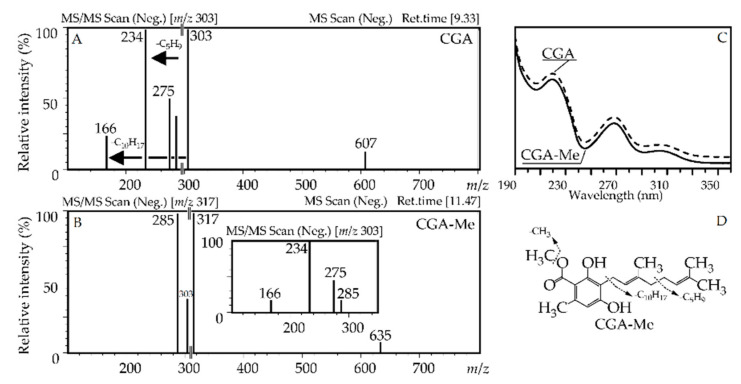
Mass spectra (**A**,**B**); negative ionization), UV patterns (**C**) of cannabigerorcinic acid (CGA) and cannabigerorcinic acid methyl ester (CGA-Me), and fragmentation way of CGA-Me (**D**).

Cannabigerorcinic acid (**120**), or 2,4-dihydroxy-6-methyl-3-[(2*E*)-3,7-dimethyl- 2,6-octadien-1-yl]-benzoic acid, was identified using the reference standard showing a specific UV pattern (λ_max_ 220, 268, 306 nm) (Figure 6). Mass spectra demonstrated loss of water (*m*/*z* 303→285), CO (*m*/*z* 303→275), and fragments of C_5_H_9_ (*m*/*z* 303→234) and C_10_H_17_ (*m*/*z* 303→166), which are characteristic for the orcinoids and cannabigerol type of phytocannabinoids [53]. Compound **120** is a typical component of *Cannabis* plants [63] and has previously been found in the CO_2_ extract of *R. adamsii* [13]. Two mono-*O*-acetates, **123** and **124**, and one di-*O*-acetate **129** of cannabigerorcinic acid showed a typical loss of one and two acetyl fragments, respectively, with mass 42 a.m.u. More polar derivatives of **120** were identified as di-*O*-hexoside (**116**) and mono-*O*-hexoside (**117**), due to the registered loss of hexosyl residues with a mass 162 a.m.u. The known analogs of cannabigerorcinic acid acetates and glycosides are still undiscovered.

Compound **79**, which dominated in the high retention time fragment of the chromatogram of SPE-2 eluate, showed a UV pattern close to **120**, however, its deprotonated ion was 14 a.m.u. higher with *m*/*z* 317 (Figure 6). The easy loss of a 14 a.m.u. fragment indicated this compound was a methyl ester of **120** (*m*/*z* 317→303), a known component of *R. adamsii* [11]. The isomeric to **79** mono-methyl esters **80**, two dimethyl esters **81** and **82**, and trimethyl ester **83** were found in *R. adamsii* for the first time, as were glycosides of cannabigerorcinic acid methyl ester, including di-*O*-hexosides (**70**, **71**), *O*-hexoside-*O*-desoxyhexoside (**72**), and *O*-hexoside (**75**), and three *O*-hexosides of cannabigerorcinic acid di-*O*-methyl ester (**76**–**78**). 

**Figure 7:**
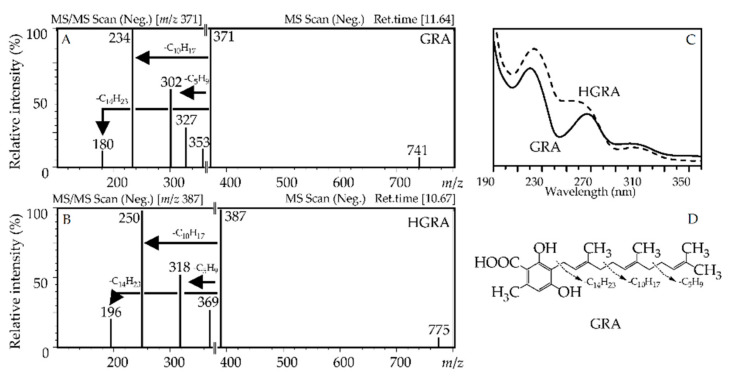
Mass spectra (**A**,**B**); negative ionization), UV patterns (**C**) of grifolic acid (GRA) and hydroxy-grifolic acid (HGRA), and fragmentation way of GRA (**D**).

Grifolic acid (**137**), or 2,4-dihydroxy-6-methyl-3-[(2*E*,6*E*)-3,7,11-trimethyl- 2,6,10-dodecatrien-1-yl]-benzoic acid, was identified by comparison with a reference standard. Grifolic acid demonstrated a UV pattern close to **120** and mass spectra indicating the presence of a longer prenyl fragment in the side chain (Figure 7); this compound has previously been detected in *Rhododendron dauricum* [64], although not in *R. adamsii*. Some esters of grifolic acid were also found in the plant as mono-*O*-methyl ester **138**, di-*O*-methyl ester **139**, and *O*-methyl ester-*O*-acetate **140**, as well di-*O*-hexoside **131** and mono-*O*-hexoside **132**, all of which remain unknown. 

Compound **130** has a lower retention time than **137** (t_R_ 10.67 vs. 11.64 min) and a 16 a.m.u. higher mass of the deprotonated ion (*m*/*z* 387 vs. 371) while the general mass spectral pattern is similar, indicating an additional hydroxyl functional group in molecule **130** (Figure 7). The difference in the UV spectrum of hydroxy-grifolic acid was that it demonstrates a shoulder-like maxima at 270 nm, in contrast to the true extreme curve of grifolic acid. The hydroxy-grifolic acid derivatives were four unknown glycosides, di-*O*-hexoside **125**, mono-*O*-hexosides **126** and **127**, and mono-*O*-pentoside **128**.

**Figure 8:**
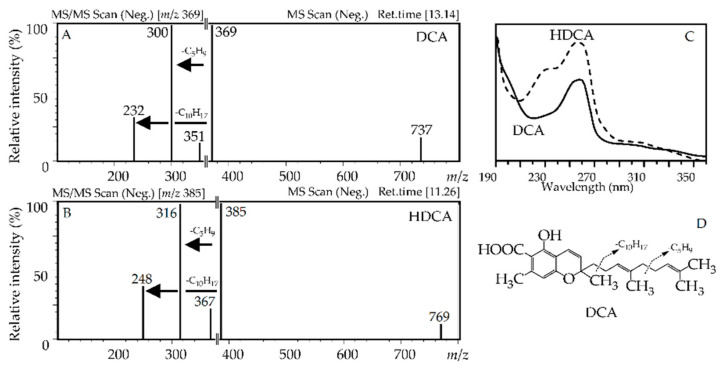
Mass spectra (**A**,**B**); negative ionization), UV patterns (**C**) of daurichromenic acid (DCA) and hydroxy-daurichromenic acid (HDCA), and fragmentation way of DCA (**D**).

Compound **141** was identified as daurichromenic acid, or 2-[(3*E*)-4,8-dimethyl-3,7-nonadien-1-yl]-5-hydroxy-2,7-dimethylchromene-6-carboxylic acid, due to its specific UV and mass spectral patterns [54], which were matched with a reference standard (Figure 8). Four esters of daurichromenic acid were described as *O*-acetate (**142**), *O*-methyl ester (**143**), *O*-methyl ester-*O*-acetate (**144**), and di-*O*-methyl ester (**145**), while two glycosides were di-*O*-hexoside (**133**) and mono-*O*-hexoside (**134**).

The *m*/*z* value of the deprotonated ion of compound **135** was 16 a.m.u. higher than that of daurichromenic acid, although the way of fragmentation was close to **141** (Figure 8). It was obvious that **135** was a hydroxy-derivative of daurichromenic acid with the additional hydroxyl group in the chromene fragment of the molecule, as indicated by the extra shortwave band in the UV spectrum. Additionally, the *O*-methyl ester of hydroxy-daurichromenic acid (**136**) was found in *R. adamsii*. All derivatives of daurichromenic acid and hydroxy-daurichromenic acid have not previously been described.

Finally, thirty-eight prenylated phenols were found in *R. adamsii*, with only three of these compounds (cannabigerorcinic acid, cannabigerorcinic acid methyl ester, and daurichromenic acid) being previously described in the plant. Existing data regarding the bioactivity of *Rhododendron* cannabinoids demonstrate anti-HIV, antiallergic, anti-inflammatory, antithrombotic, antipsychotic, anticancer, and anti-Alzheimer potential [62,65], indicating a need for further study of *R. adamsii* phytocannabinoids.

#### 3.2.6. Benzoic Acid Derivatives, Hydroxycinnamates, and Dihydrochalcones

Twenty benzoic acids of *R. adamsii* were derivatives of protocatechuic acid (**84**, **85**), vanillic/isovanillic acid (**87**–**90**), and gallic acid (**146**–**151**), including three compounds, vanillic acid 4-*O*-glucoside (**89**), gallic acid (**148**), and gallic acid *O*-methyl ester (**151**), that were identified using reference standards. Protocatechuic acid was found as di-*O*-hexoside (**84**) and mono-*O*-hexoside (**85**), vanillic/isovanillic acid as mono-*O*-hexosides (**87**, **88**, **90**), gallic acid as di-*O*-hexoside (**146**) and mono-*O*-hexoside (**147**), and gallic acid *O*-methyl ester as mono-*O*-hexosides (**150, 151**). Previously, compounds **148** and **151** have been found in *Rhododendron* plants [16,49], however, benzoic acid *O*-hexosides are still unknown in the genus.

Four hydroxycinnamates, as mono-caffeoylquinic acids, were detected in *R. adamsii* and identified with 1-*O*- (**86**), 3-*O*- (**96**), 4-*O*- (**98**), and 5-*O*-caffeoylquinic acid (**95**) after comparison with reference standards. Chlorogenic acid is the only compound previously identified in *R. kotschyi* and *R. mucronulatum* [49].

Only one dihydrochalcone phloretin (**63**) was found in the methanol eluates (SPE-2) of *R. adamsii* leaves. A previous case of the discovery of **63** in *Rhododendron* genus refers to *R. molle* [49].

#### 3.2.7. Catechins and Procyanidins

Eight catechins and five procyanidins were detected in *R. adamsii* leaves, including the reference standard identified compounds catechin (**155**), epicatechin (**159**), catechin 3-*O*-gallate (**161**), epicatechin 3-*O*-gallate (**164**), and procyanidins B_1_ (**154**), B_2_ (**158**) and C_1_ (**160**). Additional compounds were catechin/epicatechin di-*O*-hexoside (**152**) and mono-*O*-hexosides (**153, 157**), catechin/epicatechin *O*-gallate-*O*-hexoside (**156**), and catechin/epicatechin dimer *O*-gallate (**162**) and di-*O*-gallate (**163**). Catechins and procyanidins are usual components of both *Rhododendron* species and Ericaceae plants [49], with catechin/epicatechin *O*-hexosides referred to in genus for the first time.

#### 3.2.8. Chemotaxonomic Significance of *R. adamsii* Metabolites

The tribe Fragrantica E. Busch. The *Rhododendron* genus includes the Siberian species *R. adamsii* as well as the Himalayan species *R. anthopogon* D. Don, Chinese species *R. anthopogonoides* Maxim. and *R. cephalanthum* Franch., and Afghan species *R. collettianum* Aitch. & Hemsl. [66]. Despite the varying levels of knowledge of Fragrantica species chemistry, we found information regarding ursolic acid [67], epicatechin, hyperoside, and quercitrin [68] in *R. anthopogon*; quercetin, isorhamnetin, hyperoside [69], kaempferol, taxifolin, ursolic acid [70], and phytocannabinoids [71,72] in *R. anthopogonoides*; and quercetin [73] and phytocannabinoids [74] in *R. collettianum*. Flavonols of the quercetin and kaempferol type, dihydroflavonols (taxifolin), and ursolic acid have a wide distribution in the *Rhododendron* genus [49], while phytocannabinoids of orcinoids and the cannabigerol type are still rare Ericaceous compounds and could be specific markers of Fragrantica series rhododendrons.

#### 3.2.9. Seasonal Variation of *R. adamsii* Metabolites

Non-deciduous (or evergreen) plants, such as *R. adamsii*, can protect the integrity of their leaves, irrespective of environmental temperatures and season. Due to this feature, people collect the leaves of *R. adamsii* year-round, however, there is no information about the seasonal variation of metabolites. Quantification data of 171 compounds in 215 samples of *R. adamsii* leaves, collected across four seasons, demonstrated significant variability in the contents of all non-trace compounds (Table 3). Grouping the compounds into two clusters (phenolics and non-phenolics), it can be seen that the content of both clusters increased from January to July and reduced in December (Table 4). The maximal phenolics/non-phenolics level was in July (186.28/131.47 mg/g) while the lowest level was in January and March (97.26/48.95 mg/g). This means that more extractable compounds accumulate in summer samples. 

The content levels of the smaller groups of compounds conform to the same rule, with the exception of prenylated phenols. Increased levels of grifolic acid derivatives were observed in October samples, with values up to 17.53 mg/g vs. 8.35 mg/g in May samples, while cannabigerorcinic acid derivatives in the December samples reached 47.14 mg/g vs. 25.18 mg/g in July samples and daurichromenic acid derivatives in December reached 8.04 mg/g vs. 1.29 mg/g in May samples. Close variations in content were found for the flavonoid aglycones that showed the highest amounts in December samples, such as flavonol aglycones (2.54 mg/g in December vs. 0.28 mg/g in July) and flavone aglycones (1.58 mg/g in December vs. trace content in the spring and summer). Lipophilic compounds differed from the core metabolites with medium and high polarity in a seasonal variation pattern. 

The flavonoid aglycones are the usual components of leaf surface wax, which accumulates in the winter period and plays an ecophysiological function in plant development [75,76], unlike the little-known prenylated phenols of rhododendrons. In this regard, we assumed that the prenylated phenols of *R. adamsii* are the leaves’ surface components, which was confirmed after the analysis of surface diethyl ether extract. Derivatives of cannabigerorcinic acid, grifolic acid, and daurichromenic acid were detected in ether extract at a high level, whilst trace content was detected in the extract of ether-treated leaves (Figure S3). The total yield of ether extract was at a maximal in December samples (25.3% of dry leaf weight) and a minimal in July samples (11.24% of dry leaf weight) (Figure S4); this enables us to suggest that lipophilic prenylated phenols of *R. adamsii* have a protective effect in the winter period on an equal basis with other lipids covered on the leaf surface of evergreen plants [77,78,79]. 

**Figure 9:**
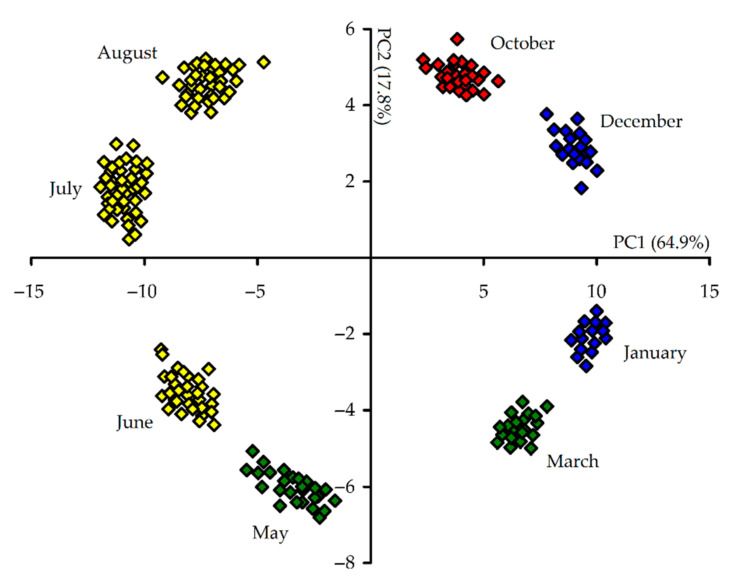
Principal component analysis (PCA) for the content of 171 compounds in 215 samples of *R. adamsii* leaves collected during various months of the year.

The results of principal component analysis (PCA) of the content of 171 compounds in 215 samples of *R. adamsii* leaves, collected in various months of the year, showed that the specific distribution of individual points on the diagram, all located on the sides of the round that means the metabolic changes occur gradually (Figure 9). 

The chemical study undertaken has demonstrated that the extractable metabolites of *R. adamsii* leaves are a very complex mixture of compounds of different nature and polarity, with significant variation depending on the season of collection. We expected to discover variable bioactivity in *R. adamsii* extracts prepared from plant materials collected during different seasons, an idea that was confirmed by our subsequent experiments.

### 3.3. Bioactivity of R. adamsii Extracts: Seasonal Changes of Antioxidant and Adaptogenic Potential

#### 3.3.1. Antioxidant Activity

Seasonal variation of the *R. adamsii* metabolome reflected varying contents of the bioactive compounds, as expected. Extracts prepared from plant material collected in four different seasons (January, May, July, and October) was inspected for antioxidant potential via nine traditional assays [80], including radical scavenging against 2,2-diphenyl-1-picrylhydrazyl radicals (DPPH^•^), 2,2’-azino-bis(3-ethylbenzothiazoline-6-sulfonic acid) radicals (ABTS^•+^), *N*,*N*-dimethyl-*p*-phenylenediamine radicals (DMPD^•+^), superoxide radicals (O_2_^•−^), hydroxyl radicals (^•^OH), and chlorine radicals (Cl^•^), as well as carotene bleaching assay, nitric oxide scavenging assay, and ferrous ions (Fe^2+^) chelating ability (Table 5). Ten selected compounds that are representatives of various metabolite groups found in *R. adamsii* were also analyzed.

Study of the antioxidant potential of *R. adamsii* extracts against artificial radicals, such as DPPH^•^, ABTS^•+^, and DMPD^•+^, demonstrated superior scavenging effects in July samples, with IC_50_ values of 3.27, 8.25, and 37.53 μg/mL, respectively. Samples collected in January were less active, with IC_50_ values of 25.37, 15.80, and 87.35 μg/mL for DPPH^•^, ABTS^•+^, and DMPD^•+^ radicals, respectively; the same parameters for Trolox, which was used as a reference compound, were 8.89, 3.02, and 53.10 μg/mL, respectively. Oxygen-radicals, as superoxide and hydroxyl radicals, were inactivated by all *R. adamsii* extracts, with the highest effectiveness observed for July samples (25.83 and 5.43 μg/mL, respectively), which showed a good protective effect in the carotene bleaching assay (12.50 μg/mL vs. 20.63 μg/mL for Trolox) and chlorine-radical scavenging assay (475.62 μg/mL vs. 1000 μg/mL for Trolox). 

The ability of *R. adamsii* extracts to scavenge nitric oxide molecules was poor (3.67 mg/mL for July sample) in comparison to Trolox (0.83 mg/mL), in contrast to the Fe^2+^-chelating ability of *R. adamsii* extract, which reached a maximum in the July sample (211.74 mg Fe^2+^/g) and minimum in the January sample (129.03 mg Fe^2+^/g), still exceeding the Trolox value (42.72 mg Fe^2+^/g). The compounds providing the most significant impact on artificial/oxygen/chlorine/nitric oxide radical scavenging ability and Fe^2+^-chelating ability were gallic acid, catechin, myricetin-3-*O*-glucoside, and quercetin-3-*O*-glucoside, which are also known as radical-scavengers [81], unlike prenylated phenols, phenol glycosides, triterpenes, and organic acids, which were inactive or at low activity levels. Thus, the high seasonal content of flavonoids and catechins is the reason why July samples of *R. adamsii* extract are the most active in all antioxidant assays studied. 

**Figure 10:**
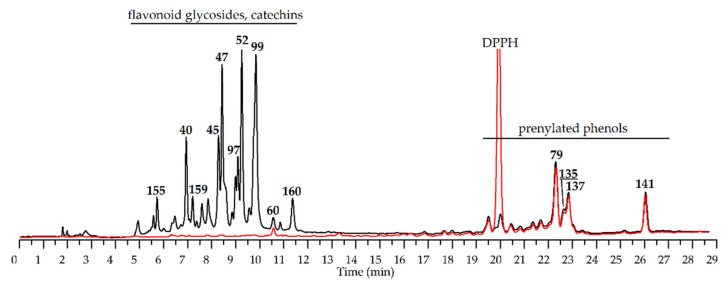
HPLC-UV chromatograms of *R. adamsii* leave total extract (July sample) before (black) and after preincubation (red) with DPPH^•^ radicals solution. The excess of DPPH^•^ radicals signed as DPPH. The basic peaks are numbered as described in Table 3.

Previously, some fungal prenylated phenols have been concluded to be medium effectiveness antioxidants, with IC_50_ values in the DPPH assay ranging from 60–80 μg/mL [82]; therefore, our results are not that surprising. The HPLC-DAD assay coupled with precolumn incubation of *R. adamsii* extract with DPPH^•^ solution showed an almost complete reduction of the flavonoid peaks area, indicating their primary activity in the free radical scavenging process (Figure 10). 

Derivatives of cannabigerorcinic, grifolic, and daurichromenic acid did not demonstrate visible changes of peak area due to weak activity. There is a clear demonstration of the important role of selected phenolic compounds (flavonoids, catechins) in the antioxidant properties of *R. adamsii* extracts.

#### 3.3.2. Adaptogenic Activity

**Figure 11:**
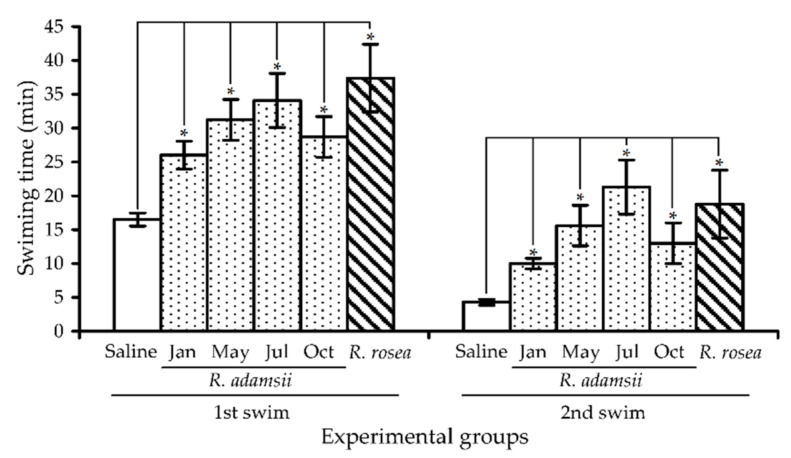
Effects of four different *R. adamsii* leaf extracts (January, May, July, and October samples) and *R. rosea* extract (50 mg/kg) on swimming time of mice in a two-step swimming to exhaustion test. * —*p* < 0.05 vs. saline group.

In further examination of the adaptogenic potential of *R. adamsii*, we analyzed the effectiveness of four leaf extracts with different seasonal origins—January, May, July, and October, in a two-step to exhaustion swimming test assessing the influence of the remedy on physical performance (first step) and endurance (second step). Animals in the control group, which received saline, showed 16.5 and 3.9 min swimming times in the 1st and 2nd steps of the test, compared to the *R. rosea* extract group used as a positive control, which showed 37.4 and 18.8 min swimming times in the two steps, respectively (Figure 11). The observed differences in the results of the *R. rosea* group compared to the saline group demonstrate stimulation of physical performance and the endurance of the animals.

Statistically significant (*p* < 0.05) increases in swimming time were observed in all experimental groups receiving *R. adamsii* leaf extracts when compared to the control group. The July group showed the best results, with values of 34.1 and 21.3 min in the first and second swimming steps, respectively, indicating that *R. adamsii* positively influences physical performance and endurance. The remaining *R. adamsii* groups demonstrated progressive decreases in swimming times when analyzing samples from the May to January groups, similar to the pattern observed for changes in the antioxidant potential of the extracts.

The two-step swimming to exhaustion test, as an exhaustive exercise, reflects on the biochemical parameters of the experimental animals. This exercise leads to fatigue and inability by the animals to perform any further active swimming movements, resulting from decreased macroergic compound content in the skeletal muscles, almost complete exhaustion of glucose concentrations in the blood and glycogen in the liver, and, accordingly, the accumulation of lactate and pyruvate [83]. Compared with intact mice, the saline group animals showed significant decreases in adenosine triphosphate (ATP; 359 vs. 71 pmol/g) and creatine phosphate (CP; 3215 vs. 937 pmol/g) in their skeletal muscles, as well as decreased glucose levels in the blood serum (9.5 vs. 1.1 mmol/L) and decreased glycogen content in the liver (23.3 vs. 5.7 mg/g), accompanied by increased levels of lactate in the skeletal muscle (3.7 vs. 8.8 μmol/kg) and pyruvic acid in the blood serum (210 vs. 1408 pg/mL) (Table 6). The expressed consumption of macroergic compounds (ATP, CP) and accumulation of acidic products in the animals points to very intense physical stress resulting in oxidative misbalance due to the accumulation of malondialdehyde (MDA; 1.8 vs. 6.7 nmol/L) and decreases catalase activity in the blood serum (11.5 vs. 6.2 mcat/L). 

A different picture emerged following similar physical activity after intake of either *R. adamsii* or *R. rosea* extracts by the animals. Both extracts, in doses of 50 mg/kg, resulted in increased working ability due to metabolic restructuring through an improvement in the energy supply of skeletal muscles, as evidenced by increases in ATP and CP contents equivalent to 1.7–2.4 times the values of the control group. Also, increased carbohydrate reserves were observed, as indicated by elevated levels of blood serum glucose and liver glycogen, while reduced levels of lactate, observed in the skeletal muscle, and pyruvate, in the blood serum, pointed to decreased levels of metabolic acidosis. The level of MDA in blood serum decreased, from 6.7 nmol/L in the control group to 2.8 nmol/L in the *R. adamsii* group and 2.3 nmol/L in the *R. rosea* group, while catalase activity levels were similar in these groups to the intact group value. Thus, under strong physical stress, *R. adamsii* demonstrated the most important properties of an adaptogenic drug, providing more economical use of the energy substrates and increasing the body’s ability to function optimally with less energy consumption [84]. 

It is worth noting that the oral administration of *R. adamsii* extract to intact mice, in the absence of physical activity, was not accompanied by pronounced changes in the analyzed parameters of carbohydrate and energy metabolism; these results meet the basic requirement of an adaptogenic drug, which should mainly work during periods of stress and provide minimal activity under normal conditions [2]. All of the foregoing indicates that *R. adamsii* leaf extract provides a positive therapeutic effect on animals during situations of strong physical stress, similar to the way known herbal adaptogens (*Rhodiola rosea*, *Eleuterococcus senticosus*, *Schizandra chinensis*) affect these animals [4]. 

## 4. Conclusions

In the situation of the global catastrophe caused by the COVID-19 epidemic, scientific findings relating to drugs that mobilize the internal reserves of protective barriers of the human body are highly relevant [85]. Adaptogenic plant drugs are considered as possible prophylactic agents for alleviating the severity of COVID-19 symptoms [86]. The expansion of the range of known plant adaptogens in this regard is an extremely important scientific mission. Thus, we can give the first detailed opinion concerning the veracity of early ethnopharmacological views of *R. adamsii* leaf extracts providing positive effects on humans as an adaptogenic remedy. Additional experiments will be necessary to understand the mechanisms of activity of *R. adamsii* extracts, as well as to elucidate their safety data. Nonetheless, it is now possible to say that Sagan Dalya tea has good prospects for medical and therapeutic use.

## Figures and Tables

**Table 1:** HPLC conditions used for metabolite separation.

Mode No, SPE Eluate (SPE Eluent)	Column	Column Temp., °C	Eluents A/B Composition	Gradient Program, %B	Flow Rate, μL/min
Mode 1, SPE-1 (H_2_O)	ProteCol™ C18 HPH125 (4.6 × 250 mm, 5 μm; Trajan Scientific Australia Pty Ltd., Ringwood, Victoria, Australia)	22	0.2% HCOOH in water/MeCN	0–1 min 5–6%, 2–5 min 6–8%, 5–8 min 8–15%, 8–15 min 15–29%, 15–20 min 29–5% B	100
Mode 2, SPE-2 (MeOH)	GLC Mastro (2.1 × 150 mm, 3 μm; Shimadzu, Kyoto, Japan)	30	0.5% HCOOH in water/0.5% HCOOH in MeCN	0–2 min 3–8%, 2–5 min 8–9%, 5–12 min 9–36%, 12–13 min 36–59%, 13–15 min 59–78%, 15–22 min 78–3%	150
Mode 3, SPE-3 (0.55% NH_3_ in MeOH)	GLC Mastro (2.1 × 150 mm, 3 μm; Shimadzu, Kyoto, Japan)	28	0.5% HCOOH in water/0.5% HCOOH in MeOH	0–1 min 5–12%, 1–3 min 12–16%, 3–7 min 16–29%, 7–11 min 29–49%, 11–15 min 49–87%, 15–25 min 87–5%	150
Mode 4, SPE-4 (DMSO)	Acclaim 120 C18 (2.1 × 150 mm, 2.2 μm; Dionex, Sunnyvale, CA, USA)	25	0.1% TFA in water/0.1% TFA in MeCN	0–3 min 0–5%, 3–8 min 5–10%, 8–15 min 8–14%, 15–17 min 14–33%, 17–22 min 33–59%, 22–30 min 59–73%, 30–40 min 73–0%	300

**Table 2:** Chemical composition and DPPH**^•^** scavenging activity of *R. adamsii* leave extracts, ± S.D.

Parameter	Solvent Type, % Methanol							
	0	20	40	50	60	70	80	100
Yield, g/100 g	12.5 ± 0.6	17.5 ± 0.8	18.5 ± 0.9	27.9 ± 1.1	30.5 ± 1.5	35.0 ± 1.7	32.5 ± 1.6	32.0 ± 1.4
Protein, mg/g	23.69 ± 0.71	11.27 ± 0.31	2.09 ± 0.06	n.d.	n.d.	n.d.	n.d.	n.d.
Lipids, mg/g	1.18 ± 0.04	10.86 ± 0.34	53.69 ± 1.61	73.14 ± 2.19	86.03 ± 2.44	183.22 ± 5.49	229.17 ± 6.87	254.12 ± 12.70
Soluble carbohydrates, mg/g	326.03 ± 8.15	308.02 ± 7.73	153.62 ± 3.94	92.14 ± 2.25	76.02 ± 1.83	43.25 ± 1.08	20.63 ± 0.51	9.35 ± 0.23
Polysaccharides, mg/g	57.60 ± 1.73	24.18 ± 0.70	3.02 ± 0.06	n.d.	n.d.	n.d.	n.d.	n.d.
Phenolics, mg/g	163.15 ± 0.48	185.69 ± 5.57	293.35 ± 8.79	253.8 ± 6.32	197.54 ± 5.82	143.20 ± 4.25	127.03 ± 3.80	108.27 ± 3.20
Flavonols, mg/g	97.86 ± 1.95	107.75 ± 2.37	138.88 ± 2.78	102.03 ± 2.50	90.64 ± 1.90	82.16 ± 1.64	74.81 ± 1.42	64.59 ± 1.29
Flavanols, mg/g	10.21 ± 0.18	12.59 ± 0.25	16.84 ± 0.31	16.09 ± 0.27	14.69 ± 0.25	10.03 ± 0.18	5.63 ± 0.11	2.29 ± 0.04
Catechins, mg/g	40.10 ± 1.00	48.52 ± 1.28	89.27 ± 2.73	88.41 ± 2.65	82.16 ± 2.46	61.01 ± 1.65	58.44 ± 1.43	55.20 ± 1.21
Procyanidins, mg/g	14.85 ± 0.52	24.32 ± 0.87	27.98 ± 0.95	26.92 ± 0.79	25.08 ± 0.75	15.41 ± 0.57	6.65 ± 0.21	3.49 ± 0.12
DPPH**^•^**, IC_50_, μg/mL	10.47 ± 0.31	7.33 ± 0.21	4.82 ± 0.14	4.93 ± 0.15	5.67 ± 0.17	14.22 ± 0.42	16.06 ± 0.48	25.81 ± 0.77

n.d.—not detected.

**Table 3:** Compounds **1**–**171** were found in *R. adamsii* leaves with their seasonal content.

No	t_R_, min ^a^	Compound ^b^ [Ref. ^c^]	[M − H]^−^, *m*/*z*	Content in Leaves ^d^, mg/g of Dry Plant Weight ± S.D.							
				January (*n* = 15)	March (*n* = 19)	May (*n* = 26)	June (*n* = 31)	July (*n* = 42)	August (*n* = 36)	October (*n* = 27)	December (*n* = 19)
		**Carbohydrates**									
1	0.62 ^I^	*O*-Hexosyl-hexose ^L^	341	42.14 ± 0.45	40.32 ± 0.36	27.32 ± 1.91	35.14 ± 2.81	39.63 ± 3.18	45.18 ± 3.27	53.18 ± 5.84	50.72 ± 5.07
2	0.76 ^I^	Hexose ^L^	179	2.11 ± 0.25	4.53 ± 0.39	11.08 ± 0.67	39.65 ± 3.55	52.19 ± 4.25	18.03 ± 1.29	10.81 ± 1.15	3.76 ± 0.38
		**Organic acids**									
3	0.92 ^I^	Malic acid ^R^	133	1.02 ± 0.08	6.36 ± 0.72	14.73 ± 0.58	18.39 ± 1.20	11.07 ± 0.22	5.39 ± 0.48	2.18 ± 0.27	1.93 ± 0.20
4	1.05 ^I^	Citric acid ^R^	191	<0.01	1.93 ± 0.15	3.75 ± 0.35	5.14 ± 0.42	3.07 ± 0.33	1.53 ± 0.10	0.93 ± 0.08	0.28 ± 0.02
5	1.26 ^I^	Tartaric acid ^R^	149	< 0.01	0.92 ± 0.06	2.53 ± 0.15	5.27 ± 0.45	4.18 ± 0.37	2.11 ± 0.14	<0.01	<0.01
6	1.42 ^I^	Succinic acid ^R^	117	<0.01	<0.01	1.09 ± 0.08	1.23 ± 0.12	1.57 ± 0.14	1.43 ± 0.10	<0.01	<0.01
7	1.51 ^I^	Fumaric acid ^R^	115	<0.01	<0.01	0.93 ± 0.07	1.14 ± 0.07	1.01 ± 0.08	0.52 ± 0.04	<0.01	<0.01
		**Simple phenolic glycosides**									
8	2.48 ^I^	Phloroglucinol di-*O*-hexoside ^L^	449	<0.01	0.22 ± 0.03	0.90 ± 0.05	0.99 ± 0.10	1.63 ± 0.14	2.35 ± 0.25	<0.01	<0.01
9	2.67 ^I^	Phloroglucinol di-*O*-hexoside ^L^	449	<0.01	<0.01	0.53 ± 0.04	0.69 ± 0.04	1.12 ± 0.10	1.97 ± 0.23	0.59 ± 0.06	<0.01
10	2.75 ^I^	Phlorin (phloroglucinol *O*-glucoside) ^R^	287	0.52 ± 0.06	1.67 ± 0.15	4.39 ± 0.48	5.63 ± 0.51	7.39 ± 1.03	6.85 ± 0.83	2.11 ± 0.18	1.45 ± 0.12
11	2.93 ^I^	Phloroglucinol di-*O*-hexoside-*O*-acetate ^L^	491	1.83 ± 0.14	1.79 ± 0.18	0.59 ± 0.05	0.73 ± 0.06	0.92 ± 0.11	1.26 ± 0.11	1.43 ± 0.10	2.03 ± 0.21
12	3.09 ^I^	Phloroglucinol di-*O*-hexoside-*O*-acetate ^L^	491	0.37 ± 0.04	0.35 ± 0.04	0.21 ± 0.01	0.25 ± 0.02	0.29 ± 0.02	0.35 ± 0.02	0.40 ± 0.03	0.35 ± 0.03
13	3.18 ^I^	Phloroglucinol di-*O*-hexoside-di-*O*-acetate ^L^	533	0.68 ± 0.07	0.42 ± 0.03	0.29 ± 0.02	0.37 ± 0.04	0.38 ± 0.06	0.55 ± 0.05	0.63 ± 0.05	0.79 ± 0.07
14	3.24 ^I^	Phloroglucinol di-*O*-hexoside-di-*O*-acetate ^L^	533	0.08 ± 0.01	<0.01	<0.01	<0.01	<0.01	<0.01	<0.01	<0.01
15	3.31 ^I^	Hydroquinone di-*O*-hexoside ^L^	433	<0.01	<0.01	0.05 ± 0.00	0.05 ± 0.00	0.07 ± 0.00	0.04 ± 0.00	<0.01	<0.01
16	3.43 ^I^	Hydroquinone di-*O*-hexoside ^L^	433	0.27 ± 0.02	0.29 ± 0.02	0.57 ± 0.06	0.61 ± 0.05	0.67 ± 0.07	0.42 ± 0.34	0.31 ± 0.03	0.30 ± 0.02
17	3.64 ^I^	Arbutin (hydroquinone *O*-glucoside) ^R^	271	<0.01	<0.01	0.09 ± 0.01	0.11 ± 0.01	0.12 ± 0.01	0.12 ± 0.01	0.05 ± 0.00	<0.01
18	3.69 ^I^	Orcinol di-*O*-hexoside ^L^	447	<0.01	<0.01	<0.01	0.14 ± 0.01	0.19 ± 0.03	0.15 ± 0.02	<0.01	<0.01
19	3.81 ^I^	Hydroquinone di-*O*-hexoside-*O*-methyl ester ^L^	447	0.05 ± 0.00	0.03 ± 0.00	0.03 ± 0.00	0.03 ± 0.00	0.04 ± 0.00	0.04 ± 0.00	0.05 ± 0.00	0.05 ± 0.00
20	3.92 ^I^	Hydroquinone *O*-hexoside-*O*-methyl ester ^L^	285	<0.01	<0.01	0.02 ± 0.00	0.02 ± 0.00	0.03 ± 0.00	0.03 ± 0.00	<0.01	<0.01
21	4.09 ^I^	Sakakin (orcinol *O*-glucoside) ^R^	285	3.14 ± 0.31	3.46 ± 0.32	4.73 ± 0.52	5.79 ± 0.46	6.18 ± 0.74	6.03 ± 0.70	5.76 ± 0.42	5.31 ± 0.50
24	4.47 ^I^	Orcinol *O*-hexoside-*O*-acetate ^L^	327	<0.01	<0.01	<0.01	0.20 ± 0.01	0.42 ± 0.04	0.20 ± 0.03	<0.01	<0.01
		**Triterpene glycosides**									
22	4.26 ^I^	Ursolic acid tri-*O*-hexoside ^L^	941	<0.01	<0.01	<0.01	0.08 ± 0.01	0.53 ± 0.06	0.59 ± 0.06	<0.01	<0.01
23	4.31 ^I^	Ursolic acid tri-*O*-hexoside ^L^	941	<0.01	<0.01	0.27 ± 0.03	0.37 ± 0.04	0.63 ± 0.05	0.72 ± 0.08	<0.01	<0.01
25	4.58 ^I^	Ursolic acid di-*O*-hexoside ^L^	779	<0.01	<0.01	<0.01	<0.01	<0.01	<0.01	<0.01	<0.01
26	4.74 ^I^	Ursolic acid di-*O*-hexoside ^L^	779	<0.01	<0.01	<0.01	<0.01	<0.01	<0.01	<0.01	<0.01
27	4.79 ^I^	Ursolic acid tri-*O*-hexoside-*O*-acetate ^L^	983	0.42 ± 0.03	0.40 ± 0.05	<0.01	<0.01	0.11 ± 0.01	0.12 ± 0.02	0.26 ± 0.02	0.31 ± 0.03
28	4.97 ^I^	Ursolic acid tri-*O*-hexoside-*O*-acetate ^L^	983	0.69 ± 0.07	0.63 ± 0.05	<0.01	0.08 ± 0.01	0.22 ± 0.02	0.25 ± 0.03	0.41 ± 0.03	0.73 ± 0.06
29	5.18 ^I^	Ursolic acid di-*O*-hexoside-*O*-acetate ^L^	821	<0.01	<0.01	<0.01	<0.01	<0.01	<0.01	<0.01	<0.01
30	5.46 ^I^	Ursolic acid *O*-hexoside ^L^	617	2.57 ± 0.31	4.85 ± 0.32	11.73 ± 1.42	15.37 ± 1.50	17.26 ± 1.83	17.54 ± 1.85	14.31 ± 1.28	3.06 ± 0.36
31	5.53 ^I^	Ursolic acid di-*O*-hexoside-di-*O*-acetate ^L^	863	<0.01	<0.01	<0.01	<0.01	<0.01	<0.01	<0.01	<0.01
32	5.74 ^I^	Ursolic acid di-*O*-hexoside-di-*O*-acetate ^L^	863	<0.01	<0.01	<0.01	<0.01	<0.01	<0.01	<0.01	<0.01
33	7.87 ^I^	Ursolic acid *O*-hexoside-*O*-acetate ^L^	659	<0.01	<0.01	<0.01	<0.01	<0.01	<0.01	<0.01	<0.01
34	8.28 ^I^	Ursolic acid *O*-hexoside-*O*-acetate ^L^	659	<0.01	<0.01	<0.01	<0.01	<0.01	<0.01	<0.01	<0.01
35	10.26 ^I^	Ursolic acid *O*-hexoside-di-*O*-acetate ^L^	701	<0.01	<0.01	<0.01	<0.01	<0.01	<0.01	<0.01	<0.01
		**Flavonols**									
		*Glycosides: myricetin derivatives*									
36	2.72 ^II^	Myricetin tri-*O*-hexoside-tri-*O*-desoxyhexoside ^L^	1241	<0.01	<0.01	<0.01	<0.01	<0.01	<0.01	<0.01	<0.01
37	2.79 ^II^	Myricetin tri-*O*-hexoside-di-*O*-desoxyhexoside ^L^	1095	<0.01	<0.01	<0.01	<0.01	<0.01	<0.01	<0.01	<0.01
46	4.09 ^II^	Myricetin di-*O*-hexoside-di-*O*-desoxyhexoside ^L^	933	<0.01	<0.01	1.53 ± 0.11	1.67 ± 0.12	1.99 ± 0.17	1.52 ± 0.12	0.35 ± 0.04	<0.01
47	4.18 ^II^	Myricetin di-*O*-hexoside-*O*-desoxyhexoside ^L^	787	<0.01	<0.01	3.63 ± 0.29	3.69 ± 0.27	4.35 ± 0.52	4.10 ± 0.32	1.76 ± 0.15	<0.01
49	4.31 ^II^	Myricetin *O*-hexoside-*O*-desoxyhexoside ^L^	625	<0.01	<0.01	<0.01	<0.01	<0.01	<0.01	<0.01	<0.01
50	4.43 ^II^	Myricetin 3-*O*-rutinoside ^R^	625	<0.01	<0.01	0.53 ± 0.04	0.79 ± 0.06	0.75 ± 0.07	0.42 ± 0.03	<0.01	<0.01
51	4.51 ^II^	Myricetin 3-*O*-galactoside ^R^	479	<0.01	<0.01	0.75 ± 0.08	1.27 ± 0.12	1.53 ± 0.16	1.42 ± 0.12	0.53 ± 0.04	<0.01
52	4.58 ^II^	Isomyricitrin (myricetin 3-*O*-glucoside) ^R^	479	7.18 ± 0.86	9.06 ± 0.89	18.67 ± 1.68	22.14 ± 2.65	25.83 ± 2.45	21.15 ± 1.90	20.63 ± 1.85	9.32 ± 0.92
56	4.97 ^II^	Myricetin *O*-pentoside ^R^	449	<0.01	<0.01	<0.01	<0.01	<0.01	<0.01	<0.01	<0.01
58	5.26 ^II^	Myricitrin (myricetin 3-*O*-rhamnoside) ^R^	463	<0.01	<0.01	0.43 ± 0.03	0.52 ± 0.06	0.63 ± 0.05	0.27 ± 0.03	<0.01	<0.01
91	3.42 ^III^	Myricetin di-*O*-hexoside-di-*O*-hexuronide ^L^	993	<0.01	<0.01	<0.01	<0.01	<0.01	<0.01	<0.01	<0.01
92	3.54 ^III^	Myricetin di-*O*-hexoside-di-*O*-hexuronide ^L^	993	<0.01	<0.01	<0.01	<0.01	<0.01	<0.01	<0.01	<0.01
93	3.74 ^III^	Myricetin di-*O*-hexoside-*O*-hexuronide ^L^	817	<0.01	<0.01	<0.01	<0.01	<0.01	<0.01	<0.01	<0.01
94	4.11 ^III^	Myricetin *O*-hexoside-di-*O*-hexuronide ^L^	831	<0.01	<0.01	<0.01	<0.01	<0.01	<0.01	<0.01	<0.01
97	4.67 ^III^	Myricetin *O*-hexoside-*O*-hexuronide ^L^	655	2.63 ± 0.31	1.37 ± 0.14	6.40 ± 0.70	7.55 ± 0.63	9.32 ± 0.74	9.07 ± 0.54	5.18 ± 0.41	4.57 ± 0.37
99	4.89 ^III^	Myricetin *O*-hexuronide ^L^	493	4.76 ± 0.30	3.22 ± 0.35	8.26 ± 0.75	10.29 ± 0.92	11.57 ± 1.23	10.83 ± 1.05	8.62 ± 0.73	5.62 ± 0.54
106	6.01 ^III^	Myricetin *O*-hexuronide-*O*-acetate ^L^	535	<0.01	<0.01	<0.01	<0.01	<0.01	<0.01	<0.01	<0.01
107	6.78 ^III^	Myricetin *O*-hexoside-di-*O*-acetate ^L^	563	<0.01	<0.01	<0.01	<0.01	<0.01	<0.01	<0.01	<0.01
108	6.92 ^III^	Myricetin *O*-hexouronide-di-*O*-acetate ^L^	577	<0.01	<0.01	<0.01	<0.01	<0.01	<0.01	<0.01	<0.01
111	7.92 ^III^	Myricetin *O*-hexoside-di-*O*-acetate ^L^	563	<0.01	<0.01	<0.01	<0.01	<0.01	<0.01	<0.01	<0.01
118	9.18 ^III^	Myricetin *O*-hexoside-tri-*O*-acetate ^L^	605	<0.01	<0.01	<0.01	<0.01	<0.01	<0.01	<0.01	<0.01
119	9.24 ^III^	Myricetin *O*-hexoside-tri-*O*-acetate ^L^	605	<0.01	<0.01	<0.01	<0.01	<0.01	<0.01	0.53 ± 0.03	0.40 ± 0.03
165	22.97 ^IV^	Myricetin tri-*O*-hexoside-di-*O*-gallate ^L^	1107	<0.01	<0.01	<0.01	<0.01	<0.01	<0.01	<0.01	<0.01
166	23.67 ^IV^	Myricetin di-*O*-hexoside-di-*O*-gallate ^L^	945	<0.01	<0.01	<0.01	<0.01	<0.01	<0.01	<0.01	<0.01
167	24.43 ^IV^	Myricetin di-*O*-hexoside-*O*-gallate ^L^	793	<0.01	<0.01	<0.01	0.14 ± 0.02	0.37 ± 0.03	0.25 ± 0.02	<0.01	<0.01
168	25.63 ^IV^	Myricetin *O*-hexoside-*O*-gallate ^L^	631	<0.01	<0.01	<0.01	<0.01	<0.01	<0.01	<0.01	<0.01
169	25.83 ^IV^	Myricetin *O*-hexoside-*O*-gallate ^L^	631	1.04 ± 0.10	0.82 ± 0.09	1.55 ± 0.17	2.80 ± 0.25	4.18 ± 0.38	3.53 ± 0.32	1.77 ± 0.18	1.52 ± 0.14
		*Glycosides: quercetin derivatives*									
38	2.97 ^II^	Quercetin tri-*O*-hexoside-di-*O*-desoxyhexoside ^L^	1079	<0.01	<0.01	<0.01	<0.01	<0.01	<0.01	<0.01	<0.01
48	4.26 ^II^	Quercetin di-*O*-hexoside-*O*-desoxyhexoside ^L^	771	<0.01	<0.01	0.93 ± 0.10	1.45 ± 0.11	1.59 ± 0.11	1.53 ± 0.12	<0.01	<0.01
53	4.74 ^II^	Rutin (quercetin 3-*O*-rutinoside) ^R^ [12]	609	<0.01	<0.01	<0.01	<0.01	<0.01	<0.01	<0.01	<0.01
54	4.78 ^II^	Hyperoside (quercetin 3-*O*-galactoside) ^R^	463	<0.01	0.26 ± 0.02	0.97 ± 0.07	1.14 ± 0.12	1.29 ± 0.14	0.92 ± 0.06	<0.01	<0.01
55	4.83 ^II^	Isoquercitrin (quercetin 3-*O*-glucoside) ^R^	463	0.29 ± 0.04	0.41 ± 0.03	1.10 ± 0.09	1.53 ± 0.12	2.83 ± 0.21	1.43 ± 0.12	0.92 ± 0.08	0.35 ± 0.03
57	5.11 ^II^	Avicularin (quercetin 3-*O*-arabinoside) ^R^ [13]	433	0.23 ± 0.02	0.20 ± 0.02	0.53 ± 0.04	0.73 ± 0.08	1.11 ± 0.10	1.12 ± 0.09	0.96 ± 0.07	0.92 ± 0.09
59	5.63 ^II^	Quercitrin (quercetin 3-*O*-rhamnoside) ^R^ [13]	433	<0.01	<0.01	0.59 ± 0.04	0.63 ± 0.07	0.65 ± 0.06	0.32 ± 0.04	<0.01	<0.01
100	5.03 ^III^	Quercetin di-*O*-hexoside-*O*-hexuronide ^L^	801	<0.01	<0.01	<0.01	<0.01	<0.01	<0.01	<0.01	<0.01
101	5.14 ^III^	Quercetin *O*-hexoside-di-*O*-hexuronide ^L^	815	<0.01	<0.01	0.50 ± 0.04	0.53 ± 0.04	0.92 ± 0.11	0.86 ± 0.12	0.27 ± 0.03	<0.01
103	5.38 ^III^	Quercetin *O*-hexoside-*O*-hexuronide ^L^	639	<0.01	<0.01	<0.01	<0.01	<0.01	<0.01	<0.01	<0.01
104	5.63 ^III^	Quercetin *O*-hexoside-*O*-hexuronide ^L^	639	<0.01	<0.01	<0.01	<0.01	<0.01	<0.01	<0.01	<0.01
105	5.69 ^III^	Miquelianin (quercetin 3-*O*-glucuronide) ^R^	477	<0.01	<0.01	<0.01	<0.01	<0.01	<0.01	<0.01	<0.01
110	7.36 ^III^	Quercetin *O*-hexuronide-*O*-acetate ^L^	519	<0.01	<0.01	<0.01	<0.01	<0.01	<0.01	0.42 ± 0.03	0.37 ± 0.03
114	8.47 ^III^	Quercetin *O*-hexuronide-di-*O*-acetate ^L^	561	<0.01	<0.01	<0.01	<0.01	<0.01	<0.01	<0.01	<0.01
115	8.69 ^III^	Quercetin *O*-hexuronide-di-*O*-acetate ^L^	561	<0.01	<0.01	<0.01	<0.01	<0.01	<0.01	<0.01	<0.01
121	9.46 ^III^	Quercetin *O*-hexoside-tri-*O*-acetate ^L^	589	<0.01	<0.01	<0.01	<0.01	<0.01	<0.01	<0.01	<0.01
122	9.53 ^III^	Quercetin *O*-hexoside-tri-*O*-acetate ^L^	589	<0.01	<0.01	<0.01	<0.01	<0.01	<0.01	<0.01	<0.01
170	26.74 ^IV^	Quercetin 3-*O*-(6’’-*O*-galloyl)-glucoside ^R^	615	0.52 ± 0.04	0.50 ± 0.05	0.83 ± 0.06	1.07 ± 0.11	1.54 ± 0.12	1.62 ± 0.14	0.93 ± 0.10	0.73 ± 0.06
171	28.02 ^IV^	Quercetin *O*-hexoside-di-*O*-gallate ^L^	767	<0.01	<0.01	<0.01	0.53 ± 0.04	0.96 ± 0.10	0.83 ± 0.09	0.21 ± 0.02	<0.01
		*Glycosides: kaempferol derivatives*									
60	5.77 ^II^	Juglanin (kaempferol 3-*O*-arabinoside) ^R^	417	<0.01	<0.01	<0.01	<0.01	<0.01	<0.01	<0.01	<0.01
61	6.29 ^II^	Afzelin (kaempferol 3-*O*-rhamnoside) ^R^	431	<0.01	<0.01	<0.01	<0.01	<0.01	<0.01	<0.01	<0.01
		*Aglycones*									
65	9.01 ^II^	Myricetin ^R^ [12]	317	0.90 ± 0.09	0.72 ± 0.08	0.39 ± 0.02	0.34 ± 0.03	0.28 ± 0.03	0.35 ± 0.04	0.83 ± 0.07	0.97 ± 0.11
**66**	9.23 ^II^	Quercetin ^R^ [12]	301	0.53 ± 0.04	0.42 ± 0.03	0.12 ± 0.01	<0.01	<0.01	<0.01	0.98 ± 0.12	1.57 ± 0.14
**69**	9.51 ^II^	Isorhamnetin ^R^	315	<0.01	<0.01	<0.01	<0.01	<0.01	<0.01	<0.01	<0.01
**73**	10.24 ^II^	Kaempferol ^R^ [13]	285	0.39 ± 0.04	<0.01	<0.01	<0.01	<0.01	<0.01	<0.01	<0.01
		**Dihydroflavonols**									
		*Dihydromyricetin derivatives*									
**39**	3.22 ^II^	Dihydromyricetin di-*O*-hexoside ^L^	643	<0.01	<0.01	<0.01	<0.01	<0.01	<0.01	<0.01	<0.01
**40**	3.45 ^II^	Dihydromyricetin *O*-hexoside ^L^	481	1.53 ± 0.16	1.48 ± 0.17	2.73 ± 0.21	3.11 ± 0.43	3.52 ± 0.47	3.18 ± 0.40	2.39 ± 0.18	2.01 ± 0.20
		*Dihydroquercetin derivatives*									
**41**	3.52 ^II^	Dihydroquercetin di-*O*-hexoside-di-*O*-desoxyhexoside ^L^	919	<0.01	<0.01	<0.01	<0.01	<0.01	<0.01	<0.01	<0.01
**42**	3.64 ^II^	Dihydroquercetin di-*O*-hexoside-*O*-desoxyhexoside ^L^	773	<0.01	<0.01	<0.01	<0.01	<0.01	<0.01	<0.01	<0.01
**43**	3.72 ^II^	Dihydroquercetin *O*-hexoside-*O*-desoxyhexoside ^L^	611	<0.01	<0.01	<0.01	<0.01	<0.01	<0.01	<0.01	<0.01
**44**	3.78 ^II^	Dihydroquercetin *O*-hexoside ^L^	465	<0.01	<0.01	<0.01	<0.01	<0.01	<0.01	<0.01	<0.01
**45**	3.83 ^II^	Astilbin (=dihydroquercetin-3-*O*-rhamnoside) ^R^	449	4.32 ± 0.51	5.27 ± 0.57	5.29 ± 0.53	6.18 ± 0.43	7.11 ± 0.78	6.53 ± 0.97	5.12 ± 0.71	5.06 ± 0.75
**62**	8.25 ^II^	Dihydroquercetin (taxifloin) ^R^ [12]	303	2.35 ± 0.16	1.58 ± 0.14	0.27 ± 0.04	0.35 ± 0.04	0.32 ± 0.03	0.41 ± 0.03	0.86 ± 0.09	1.43 ± 0.12
**102**	5.26 ^III^	Dihydroquercetin *O*-hexuronide ^L^	479	<0.01	<0.01	<0.01	<0.01	<0.01	<0.01	<0.01	<0.01
**109**	7.22 ^III^	Dihydroquercetin *O*-hexuronide-*O*-acetate ^L^	521	<0.01	<0.01	<0.01	<0.01	<0.01	<0.01	<0.01	<0.01
**112**	8.14 ^III^	Dihydroquercetin *O*-hexuronide-di-*O*-acetate ^L^	563	<0.01	<0.01	<0.01	<0.01	<0.01	<0.01	0.57 ± 0.06	0.62 ± 0.06
**113**	8.26 ^III^	Dihydroquercetin *O*-hexuronide-di-*O*-acetate ^L^	563	<0.01	<0.01	<0.01	<0.01	<0.01	<0.01	<0.01	<0.01
		*Dihydrokaempferol derivatives*									
**64**	8.83 ^II^	Dihydrokaempferol (aromadendrin) ^R^	287	0.11 ± 0.02	<0.01	<0.01	<0.01	<0.01	<0.01	<0.01	0.14 ± 0.02
		**Flavones**									
**67**	9.27 ^II^	Luteolin ^R^	285	<0.01	<0.01	<0.01	<0.01	<0.01	<0.01	<0.01	<0.01
**68**	9.37 ^II^	Apigenin ^R^	269	<0.01	<0.01	<0.01	<0.01	<0.01	<0.01	<0.01	<0.01
**74**	10.48 ^II^	Farrerol ^R^ [13]	299	0.52 ± 0.06	<0.01	<0.01	<0.01	<0.01	<0.01	0.62 ± 0.05	1.58 ± 0.14
		**Prenylared phenols**									
		*Cannabigerorcinic acid derivatives*									
**70**	9.76 ^II^	Cannabigerorcinic acid *O*-methyl ester di-*O*-hexoside ^L^	641	<0.01	<0.01	2.35 ± 0.28	2.53 ± 0.31	2.11 ± 0.27	2.53 ± 0.22	1.03 ± 11	0.58 ± 0.04
**71**	9.93 ^II^	Cannabigerorcinic acid *O*-methyl ester di-*O*-hexoside ^L^	641	<0.01	<0.01	0.62 ± 0.05	0.69 ± 0.06	0.50 ± 0.04	0.31 ± 0.02	<0.01	<0.01
72	10.04 ^II^	Cannabigerorcinic acid *O*-methyl ester *O*-hexoside-*O*-desoxyhexoside ^L^	625	<0.01	<0.01	0.95 ± 0.10	1.14 ± 0.12	1.16 ± 0.14	0.73 ± 0.08	<0.01	<0.01
75	10.63 ^II^	Cannabigerorcinic acid methyl ester *O*-hexoside ^L^	479	1.43 ± 0.12	1.20 ± 0.10	1.93 ± 0.22	2.35 ± 0.25	2.30 ± 0.23	2.04 ± 0.20	2.56 ± 0.24	2.33 ± 0.20
76	10.97 ^II^	Cannabigerorcinic acid di-*O*-methyl ester *O*-hexoside ^L^	493	<0.01	<0.01	<0.01	<0.01	<0.01	<0.01	<0.01	<0.01
77	11.05 ^II^	Cannabigerorcinic acid di-*O*-methyl ester *O*-hexoside ^L^	493	<0.01	<0.01	<0.01	<0.01	<0.01	<0.01	<0.01	<0.01
78	11.22 ^II^	Cannabigerorcinic acid di-*O*-methyl ester *O*-hexoside ^L^	493	<0.01	<0.01	<0.01	<0.01	<0.01	<0.01	<0.01	<0.01
79	11.47 ^II^	Cannabigerorcinic acid *O*-methyl ester ^L^	317	35.16 ± 3.57	32.03 ± 3.28	18.35 ± 2.14	20.39 ± 2.24	19.03 ± 1.92	25.76 ± 2.06	36.18 ± 3.25	39.92 ± 4.02
80	11.52 ^II^	Cannabigerorcinic acid *O*-methyl ester ^L^	317	0.14 ± 0.02	<0.01	<0.01	<0.01	<0.01	<0.01	<0.01	0.24 ± 0.02
81	11.74 ^II^	Cannabigerorcinic acid di-*O*-methyl ester ^L^	331	0.10 ± 0.02	<0.01	<0.01	<0.01	<0.01	<0.01	<0.01	0.10 ± 0.01
82	12.04 ^II^	Cannabigerorcinic acid di-*O*-methyl ester ^L^	331	0.20 ± 0.02	<0.01	<0.01	<0.01	<0.01	<0.01	<0.01	0.18 ± 0.02
83	13.15 ^II^	Cannabigerorcinic acid tri-*O*-methyl ester ^L^	345	0.18 ± 0.03	<0.01	<0.01	<0.01	<0.01	<0.01	<0.01	0.27 ± 0.03
116	8.92 ^III^	Cannabigerorcinic acid di-*O*-hexoside ^L^	627	<0.01	<0.01	<0.01	<0.01	<0.01	<0.01	<0.01	<0.01
117	9.01 ^III^	Cannabigerorcinic acid *O*-hexoside ^L^	465	0.40 ± 0.04	<0.01	<0.01	<0.01	<0.01	<0.01	0.92 ± 0.11	0.86 ± 0.09
120	9.33 ^III^	Cannabigerorcinic acid ^R^ [13]	303	0.63 ± 0.07	0.62 ± 0.08	0.37 ± 0.04	0.21 ± 0.02	0.08 ± 0.00	0.21 ± 0.03	0.95 ± 0.11	0.99 ± 0.11
123	9.72 ^III^	Cannabigerorcinic acid *O*-acetate ^L^	345	0.34 ± 0.03	0.19 ± 0.02	<0.01	<0.01	<0.01	<0.01	0.42 ± 0.03	0.40 ± 0.03
124	9.81 ^III^	Cannabigerorcinic acid di-*O*-acetate ^L^	387	1.05 ± 0.09	0.86 ± 0.07	<0.01	<0.01	<0.01	0.38 ± 0.04	1.53 ± 0.14	1.27 ± 0.11
129	10.40 ^III^	Cannabigerorcinic acid tri-*O*-acetate ^L^	429	<0.01	<0.01	<0.01	<0.01	<0.01	<0.01	<0.01	<0.01
		*Grifolic acid derivatives*									
125	9.93 ^III^	Hydroxy-grifolic acid di-*O*-hexoside ^L^	711	<0.01	<0.01	<0.01	<0.01	<0.01	0.53 ± 0.06	0.62 ± 0.05	0.35 ± 0.04
126	9.98 ^III^	Hydroxy-grifolic acid *O*-hexoside ^L^	549	0.42 ± 0.04	0.27 ± 0.04	1.22 ± 0.10	1.53 ± 0.16	2.14 ± 0.19	2.53 ± 0.22	1.67 ± 0.14	0.93 ± 0.10
127	10.09 ^III^	Hydroxy-grifolic acid *O*-hexoside ^L^	549	0.97 ± 0.11	0.53 ± 0.04	1.09 ± 0.12	2.75 ± 0.24	3.10 ± 0.31	3.16 ± 0.28	2.39 ± 0.22	1.86 ± 0.16
128	10.27 ^III^	Hydroxy-grifolic acid *O*-pentoside ^L^	519	0.11 ± 0.02	<0.01	<0.01	0.20 ± 0.01	0.63 ± 0.04	1.45 ± 0.10	0.92 ± 0.11	0.53 ± 0.04
130	10.67 ^III^	Hydroxy-grifolic acid ^L^	387	3.15 ± 0.40	3.01 ± 0.39	1.86 ± 0.20	1.04 ± 0.11	1.59 ± 0.14	3.67 ± 0.34	4.18 ± 0.39	4.50 ± 0.48
131	10.75 ^III^	Grifolic acid di-*O*-hexoside ^L^	695	<0.01	<0.01	<0.01	<0.01	<0.01	<0.01	<0.01	<0.01
132	10.86 ^III^	Grifolic acid *O*-hexoside ^L^	533	<0.01	<0.01	<0.01	<0.01	0.27 ± 0.03	0.50 ± 0.03	0.42 ± 0.04	0.22 ± 0.03
137	11.64 ^III^	Grifolic acid ^R^	371	7.09 ± 0.67	6.59 ± 0.65	4.18 ± 0.42	3.62 ± 0.40	5.73 ± 0.52	5.62 ± 0.57	7.33 ± 0.69	7.56 ± 0.73
138	12.72 ^III^	Grifolic acid *O*-methyl ester ^L^	385	<0.01	<0.01	<0.01	<0.01	<0.01	<0.01	<0.01	<0.01
139	12.81 ^III^	Grifolic acid di-*O*-methyl ester ^L^	399	<0.01	<0.01	<0.01	<0.01	<0.01	<0.01	<0.01	<0.01
140	12.92 ^III^	Grifolic acid *O*-methyl ester-*O*-acetate ^L^	427	<0.01	<0.01	<0.01	<0.01	<0.01	<0.01	<0.01	<0.01
		*Daurichromenic acid derivatives*									
133	10.86 ^III^	Daurichromenic acid di-*O*-hexoside ^L^	693	<0.01	<0.01	<0.01	<0.01	<0.01	<0.01	<0.01	<0.01
134	11.06 ^III^	Daurichromenic acid *O*-hexoside ^L^	531	<0.01	<0.01	<0.01	<0.01	<0.01	<0.01	<0.01	<0.01
135	11.26 ^III^	Hydroxy-daurichromenic acid ^L^	385	3.57 ± 0.33	3.02 ± 0.31	0.25 ± 0.03	0.77 ± 0.08	0.93 ± 0.11	2.09 ± 0.21	2.59 ± 0.31	3.82 ± 0.35
136	11.43 ^III^	Hydroxy-daurichromenic acid *O*-methyl ester ^L^	399	1.63 ± 0.17	1.42 ± 0.12	<0.01	<0.01	0.50 ± 0.04	0.84 ± 0.07	1.42 ± 0.10	1.53 ± 0.14
141	13.14 ^III^	Daurichromenic acid ^R^ [13]	369	2.30 ± 0.21	1.93 ± 0.20	1.04 ± 0.09	1.27 ± 0.14	1.53 ± 0.14	2.07 ± 0.17	2.56 ± 0.22	2.69 ± 0.25
142	13.42 ^III^	Daurichromenic acid *O*-acetate ^L^	411	<0.01	<0.01	<0.01	<0.01	<0.01	<0.01	<0.01	<0.01
143	13.58 ^III^	Daurichromenic acid *O*-methyl ester ^L^	383	<0.01	<0.01	<0.01	<0.01	<0.01	<0.01	<0.01	<0.01
144	13.74 ^III^	Daurichromenic acid *O*-methyl ester-*O*-acetate ^L^	425	<0.01	<0.01	<0.01	<0.01	<0.01	<0.01	<0.01	<0.01
145	14.23 ^III^	Daurichromenic acid di-*O*-methyl ester ^L^	397	<0.01	<0.01	<0.01	<0.01	<0.01	<0.01	<0.01	<0.01
		**Benzoic acid derivatives**									
84	0.68 ^III^	Protocatechuic acid di-*O*-hexoside ^L^	477	<0.01	<0.01	<0.01	0.14 ± 0.01	0.53 ± 0.04	0.63 ± 0.05	0.21 ± 0.02	<0.01
85	0.89 ^III^	Protocatechuic acid *O*-hexoside ^L^	315	<0.01	<0.01	<0.01	<0.01	0.23 ± 0.02	<0.01	<0.01	<0.01
87	2.71 ^III^	Vanillic/isovanillic acid *O*-hexoside ^L^	329	<0.01	<0.01	<0.01	<0.01	<0.01	<0.01	<0.01	<0.01
88	2.76 ^III^	Vanillic/isovanillic acid *O*-hexoside ^L^	329	<0.01	<0.01	<0.01	<0.01	<0.01	<0.01	<0.01	<0.01
89	2.81 ^III^	Vanillic acid 4-*O*-glucoside ^R^	329	<0.01	<0.01	0.53 ± 0.06	1.90 ± 0.16	2.51 ± 0.21	2.07 ± 0.20	0.95 ± 0.10	0.27 ± 0.03
90	2.95 ^III^	Vanillic/isovanillic acid *O*-hexoside ^L^	329	<0.01	<0.01	<0.01	<0.01	<0.01	<0.01	<0.01	<0.01
146	2.70 ^IV^	Gallic acid di-*O*-hexoside ^L^	493	<0.01	<0.01	<0.01	<0.01	<0.01	<0.01	<0.01	<0.01
147	3.27 ^IV^	Gallic acid *O*-hexoside ^L^	331	<0.01	<0.01	0.67 ± 0.04	1.53 ± 0.12	2.11 ± 0.19	1.83 ± 0.17	0.84 ± 0.06	<0.01
148	3.94 ^IV^	Gallic acid ^R^	169	0.26 ± 0.03	<0.01	1.72 ± 0.14	2.73 ± 0.31	4.37 ± 0.48	4.20 ± 0.45	2.63 ± 0.25	1.15 ± 0.10
149	4.32 ^IV^	Gallic acid *O*-methyl ester *O*-hexoside ^L^	345	<0.01	<0.01	<0.01	<0.01	<0.01	<0.01	<0.01	<0.01
150	4.91 ^IV^	Gallic acid *O*-methyl ester *O*-hexoside ^L^	345	<0.01	<0.01	<0.01	<0.01	<0.01	<0.01	<0.01	<0.01
151	5.60 ^IV^	Gallic acid *O*-methyl ester ^R^	183	<0.01	<0.01	<0.01	<0.01	<0.01	<0.01	<0.01	<0.01
		**Hydroxycinnamates**									
86	2.42 ^III^	1-*O*-Caffeoylquinic acid ^R^	353	<0.01	<0.01	<0.01	<0.01	<0.01	<0.01	<0.01	<0.01
95	4.26 ^III^	5-*O*-Caffeoylquinic acid ^R^	353	<0.01	<0.01	0.70 ± 0.06	0.82 ± 0.06	0.97 ± 0.10	0.52 ± 0.06	0.30 ± 0.02	0.08 ± 0.00
96	4.51 ^III^	3-*O*-Caffeoylquinic acid ^R^	353	<0.01	<0.01	<0.01	<0.01	<0.01	<0.01	<0.01	<0.01
98	4.72 ^III^	4-*O*-Caffeoylquinic acid ^R^	353	<0.01	<0.01	<0.01	<0.01	<0.01	<0.01	<0.01	<0.01
		**Catechins**									
152	7.82 ^IV^	Catechin/epicatechin di-*O*-hexoside ^L^	613	<0.01	<0.01	<0.01	<0.01	0.26 ± 0.02	<0.01	<0.01	<0.01
153	8.51 ^IV^	Catechin/epicatechin *O*-hexoside ^L^	451	<0.01	<0.01	<0.01	0.27 ± 0.02	0.58 ± 0.05	0.52 ± 0.04	0.03 ± 0.00	<0.01
155	9.82 ^IV^	Catechin ^R^	289	8.63 ± 0.85	7.16 ± 0.63	9.35 ± 1.02	10.22 ± 1.07	15.23 ± 1.40	15.39 ± 1.45	12.82 ± 1.14	10.04 ± 0.93
156	10.76 ^IV^	Catechin/epicatechin *O*-gallate-*O*-hexoside ^L^	603	<0.01	<0.01	0.02 ± 0.00	0.11 ± 0.01	0.35 ± 0.03	0.30 ± 0.02	<0.01	<0.01
157	12.15 ^IV^	Catechin/epicatechin *O*-hexoside ^L^	451	<0.01	<0.01	<0.01	<0.01	0.14 ± 0.02	0.10 ± 0.02	<0.01	<0.01
159	13.54 ^IV^	Epicatechin ^R^	289	0.86 ± 0.10	0.53 ± 0.04	1.10 ± 0.09	1.57 ± 0.16	1.54 ± 0.14	1.95 ± 0.20	1.26 ± 0.10	0.94 ± 0.09
161	19.67 ^IV^	Catechin 3-*O*-gallate ^R^	441	0.02 ± 0.00	<0.01	0.37 ± 0.04	0.39 ± 0.04	0.92 ± 0.08	0.95 ± 0.07	0.42 ± 0.03	0.11 ± 0.01
164	22.26 ^IV^	Epicatechin 3-*O*-gallate ^R^	441	<0.01	<0.01	<0.01	<0.01	<0.01	<0.01	<0.01	<0.01
		**Procyanidins**									
154	9.03 ^IV^	Procyanidin B_1_ ^R^	577	0.95 ± 0.08	0.73 ± 0.08	1.14 ± 0.10	2.06 ± 0.018	2.89 ± 0.25	2.73 ± 0.26	1.39 ± 0.14	1.22 ± 0.10
158	13.02 ^IV^	Procyanidin B_2_ ^R^	577	<0.01	<0.01	<0.01	<0.01	<0.01	<0.01	<0.01	<0.01
160	15.51 ^IV^	Procyanidin C_1_ ^R^	865	0.72 ± 0.06	0.63 ± 0.06	0.99 ± 0.10	1.27 ± 0.14	1.37 ± 0.14	1.27 ± 0.11	1.02 ± 0.09	0.83 ± 0.09
162	20.52 ^IV^	Catechin/epicatechin dimer *O*-gallate ^L^	729	<0.01	<0.01	<0.01	<0.01	<0.01	<0.01	<0.01	<0.01
163	21.48 ^IV^	Catechin/epicatechin dimer di-*O*-gallate ^L^	881	<0.01	<0.01	<0.01	<0.01	<0.01	<0.01	<0.01	<0.01
		**Dihydrochalcones**									
63	8.68 ^II^	Phloretin ^R^	273	0.18 ± 0.02	<0.01	<0.01	<0.01	<0.01	<0.01	0.23 ± 0.02	0.29 ± 0.03

^a^ Chromatographic conditions: ^I^—mode 1; ^II^—mode 2; ^III^—mode 3; ^IV^—mode 4. ^b^ Compound identification was based on comparison of retention time, UV and MS spectral data with reference standard (^R^) or interpretation of UV and MS spectral data and comparison with literature data (^L^). ^c^ In square brackets—reference for known data of compound presence in *R. adamsii*. ^d^ Content in *R. adamsii* leaves collected in various months (from January to December). *n*—number of plant samples used for analysis.

**Table 4:** Seasonal variation of the total content of compound groups in *R. adamsii* leaves, mg/g of dry plant weight.

Group of Compounds	Content, mg/g							
	January	March	May	June	July	August	October	December
Total carbohydrates	44.25	44.85	38.40	74.79	91.82	63.21	63.99	54.48
Total organic acids	1.02	9.21	23.03	31.17	20.90	10.98	3.11	2.21
Total simple phenol glycosides	6.94	8.23	12.40	15.41	19.45	20.36	11.33	10.28
incl. phloroglucinol derivatives	3.48	4.45	6.91	8.66	11.73	13.33	5.16	4.62
incl. hydroquinone derivatives	0.32	0.32	0.76	0.82	0.93	0.65	0.41	0.35
incl. orcinol derivatives	3.14	3.46	4.73	5.93	6.79	6.38	5.76	5.31
Total triterpene glycosides	3.68	5.88	12.00	15.90	18.75	19.22	14.98	4.10
Total flavonols	18.47	16.98	47.54	58.81	71.69	61.54	45.19	26.34
incl. glycosides, myricetin derivatives	15.61	14.47	41.75	50.86	60.52	52.56	39.37	21.43
incl. glycosides, quercetin derivatives	1.04	1.37	5.45	7.61	10.89	8.63	3.71	2.37
incl. glycosides, kaempferol derivatives	<0.01	<0.01	<0.01	<0.01	<0.01	<0.01	<0.01	<0.01
incl. aglycones	1.82	1.14	0.51	0.34	0.28	0.35	1.81	2.54
Total dihydroflavonols	8.31	8.33	8.29	9.64	10.95	10.12	8.94	9.26
incl. dihydromyricetin derivatives	1.53	1.48	2.73	3.11	3.52	3.18	2.39	2.01
incl. dihydroquercetin derivatives	6.67	6.85	5.56	6.53	7.43	6.94	6.55	7.11
incl. dihydrokaempferol derivatives	0.11	<0.01	<0.01	<0.01	<0.01	<0.01	<0.01	0.14
Total flavones	0.52	<0.01	<0.01	<0.01	<0.01	<0.01	0.62	1.58
Total prenylated phenols	58.87	51.67	34.21	38.49	41.60	54.42	67.69	71.13
incl. cannabigerorcinic acid derivatives	39.63	34.90	24.57	27.31	25.18	31.96	43.59	47.14
incl. grifolic acid derivatives	11.74	10.40	8.35	9.14	13.46	17.46	17.53	15.95
incl. daurichromenic acid derivatives	7.50	6.37	1.29	2.04	2.96	5.00	6.57	8.04
Total benzoic acid derivatives	0.26	<0.01	2.92	6.30	9.75	8.73	4.63	1.42
Total hydroxycinnamates	<0.01	<0.01	0.70	0.82	0.97	0.52	0.30	0.08
Total catechins	9.51	7.69	10.84	12.56	19.02	19.21	14.53	11.17
Total procyanidins	1.67	1.36	2.13	3.33	4.26	4.00	2.41	2.05
Total dihydrochalcones	0.18	<0.01	<0.01	<0.01	<0.01	<0.01	0.23	0.29
Total phenolics	107.66	97.26	126.04	153.03	186.28	184.97	159.70	136.77
Total non-phenolics	48.95	59.94	73.43	121.86	131.47	93.41	82.08	60.79
Total phenolics/non-phenolics	156.61	157.20	199.47	274.89	317.75	278.38	241.78	197.56

**Table 5:** Antioxidant activity of *R. adamsii* extracts and selected compounds.

Extract (Collection Month), Compound	DPPH^• b^	ABTS^•+ b^	DMPD^•+ b^	O_2_^•− b^	OH^• b^	CBA ^b^	Cl^• c^	NO ^d^	FeCA ^e^
*R. adamsii* extract (January)	25.37 ± 0.68	15.80 ± 0.31	87.35 ± 2.53	82.11 ± 2.46	59.73 ± 1.79	30.62 ± 1.29	263.93 ± 6.55	<5	129.03 ± 5.12
*R. adamsii* extract (May)	9.82 ± 0.21	10.54 ± 0.21	53.62 ± 1.61	52.69 ± 1.54	15.25 ± 0.42	15.35 ± 0.61	408.34 ± 10.26	4.05 ± 0.19	193.55 ± 7.63
*R. adamsii* extract (July)	3.27 ± 0.06	8.25 ± 0.16	37.53 ± 1.12	25.83 ± 0.77	5.43 ± 0.16	12.50 ± 0.53	475.62 ± 11.89	3.67 ± 0.16	211.74 ± 8.44
*R. adamsii* extract (October)	12.62 ± 0.31	12.32 ± 0.24	63.82 ± 1.99	49.63 ± 1.45	26.82 ± 0.73	27.09 ± 1.08	378.21 ± 9.40	4.89 ± 0.22	173.62 ± 6.90
Malic acid	>100	>100	>100	>200	>100	>200	<1	<5	<1
Phlorin	52.06 ± 1.63	>100	>100	>200	>100	>200	4.27 ± 0.08	<5	<1
Ursolic acid	>100	>100	>100	>200	>100	>200	<1	<5	<1
Myricetin-3-*O*-glucoside	5.83 ± 0.12	2.35 ± 0.04	18.89 ± 0.56	22.17 ± 0.66	3.81 ± 0.10	12.27 ± 0.47	893.57 ± 17.85	1.07 ± 0.04	70.52 ± 2.11
Quercetin-3-*O*-glucoside	9.36 ± 0.18	5.72 ± 0.11	62.65 ± 1.86	73.62 ± 2.21	12.63 ± 0.39	35.64 ± 1.40	569.21 ± 11.38	2.35 ± 0.09	62.04 ± 1.82
Cannabigerorcinic acid	>100	>100	>100	>200	>100	89.63 ± 3.59	25.63 ± 0.50	<5	23.12 ± 0.69
Grifolic acid	>100	>100	>100	>200	>100	124.18 ± 4.96	18.04 ± 0.32	<5	15.60 ± 0.41
Daurichromenic acid	>100	>100	>100	>200	>100	93.52 ± 3.74	22.57 ± 0.45	<5	12.09 ± 0.34
Gallic acid	1.53 ± 0.03	0.86 ± 0.02	22.45 ± 0.67	20.14 ± 0.58	9.57 ± 0.29	5.92 ± 0.23	1267.02 ± 25.27	0.97 ± 0.03	157.12 ± 4.83
Catechin	3.02 ± 0.06	1.41 ± 0.03	20.39 ± 0.60	43.10 ± 1.25	7.73 ± 0.23	26.84 ± 1.07	853.14 ± 17.06	1.56 ± 0.06	75.14 ± 2.20
Trolox ^a^	8.89 ± 0.15	3.02 ± 0.06	53.10 ± 1.59	90.63 ± 2.40	10.25 ± 0.26	20.63 ± 0.82	1000	0.83 ± 0.03	42.72 ± 1.26

^a^ Reference compound; ^b^ IC_50_, μg/mL; ^c^ mg Trolox eq./g; ^d^ IC_50_, mg/mL; ^e^ mg Fe^2+^/g.

**Table 6:** Influence of *R. adamsii* leaf extract (July sample, dose 50 mg/kg) and *R. rosea* extract (dose 50 mg/kg) on biochemical parameters of the skeletal muscles, blood serum, and liver of mice after the two-step swimming test.

Experimental Group	Skeletal Muscles			Blood Serum				Liver
	ATP, pmol/g	Creatine Phosphate, pmol/g	Lactate, μmol/kg	Pyruvic Acid, pg/mL	Glucose, mmol/L	MDA, nmol/L	Catalase, mcat/L	Glycogen, mg/g
Saline, without test (intact)	359 ± 71 *	3215 ± 160 *	3.7 ± 0.2 *	210 ± 57 *	9.5 ± 1.5 *	1.8 ± 0.1 *	11.5 ± 0.7 *	23.3 ± 1.1 *
Saline, after test (control)	71 ± 17	937 ± 53	8.8 ± 0.6	1408 ± 281	1.1 ± 0.2	6.7 ± 0.5	6.2 ± 0.5	5.7 ± 0.3
*R. adamsii*, without test	325 ± 58 *	3107 ± 156 *	3.7 ± 0.2 *	215 ± 55 *	9.7 ± 1.7 *	1.8 ± 0.1 *	11.5 ± 0.7 *	22.9 ± 1.1 *
*R. adamsii*, after test	143 ± 44 *	1631 ± 98 *	5.2 ± 0.3 *	806 ± 145 *	3.3 ± 0.5 *	2.8 ± 0.2 *	10.0 ± 0.8 *	12.8 ± 0.8 *
*R. rosea*, without test	337 ± 60 *	3163 ± 142 *	3.6 ± 0.2 *	203 ± 48 *	9.2 ± 1.1 *	1.8 ± 0.1 *	11.4 ± 0.7 *	23.5 ± 1.2 *
*R. rosea*, after test	173 ± 36 *	1986 ± 107 *	4.3 ± 0.3 *	706 ± 204 *	3.7 ± 0.7 *	2.3 ± 0.2 *	10.2 ± 0.8 *	12.0 ± 0.7 *

*—*p* < 0.05 vs. control group.

## Data Availability

Data is contained within the article.

## Supplementary Materials

The following are available online at https://www.mdpi.com/article/10.3390/antiox10060863/s1, Table S1: Reference standards used for the qualitative and quantitative analysis by HPLC-DAD-ESI-tQ-MS and HPLC-UV assays, Table S2: Regression equations, correlation coefficients, standard deviation, limits of detection, limits of quantification and linear ranges for 55 reference standards, Table S3: Retention times, UV- and ESI-MS spectral data of compounds 1–171 found in *Rhododendron adamsii*, Table S4: UV-spectral patterns of compounds found in *R. adamsii*, Figure S1: High-performance anion-exchange chromatography with photodiode detection chromatograms of free carbohydrates of *Rhododendron adamsii* leaves and reference standards, Figure S2: High-performance liquid chromatography with photodiode detection chromatograms of triterpenic acids of *R. adamsii* leaves and reference standards, Figure S3: HPLC-PDA chromatograms of ether and methanolic extracts of fresh *R. adamsii* leaves, Figure S4: Yield of ether extract from *R. adamsii* leaves collected in various months.
