# Supplementary material for: Sagan Dalya Tea, a New “Old” Probable Adaptogenic Drug: Metabolic Characterization and Bioactivity Potentials of Rhododendron adamsii Leaves
Source: Antioxidants (Basel). 2021 May 27;10(6):863. doi: 10.3390/antiox10060863 (PMC8227344; doi:10.3390/antiox10060863)
Supplement: Supplementary file 1 [file antioxidants-10-00863-s001.zip › antioxidants-1242661-supplementary.pdf]

antioxidants

MDPI

Supplementary Material

Sagan Dalya Tea, a New "Old" Probable Adaptogenic Drug: Metabolic Characterization and Bioactivity Potentials of *Rhododendron adamsii* Leaves

Daniil N. Olennikov [1,*], Vyacheslav M. Nikolaev [2] and Nadezhda K. Chirikova [3]

[1] Laboratory of Medical and Biological Research, Institute of General and Experimental Biology, Siberian Division, Russian Academy of Science, 6 Sakh'yanovoy Street, 670047 Ulan-Ude, Russia
[2] Department of the Adaptation Mechanisms Study, Yakutsk Scientific Center of Complex Medical Problems, 6/3 Yaroslavsky Street, 677000 Yakutsk, Russia; nikolaev1126@mail.ru
[3] Department of Biology, Institute of Natural Sciences, North-Eastern Federal University, 58 Belinsky Street, 677027 Yakutsk, Russia; hofnung@mail.ru

Citation: Olennikov, D.N.; Nikolaev, V.M.; Chirikova, N.K. Sagan Dalya Tea, a New "Old" Probable Adaptogenic Drug: Metabolic Characterization and Bioactivity Potentials of *Rhododendron adamsii* Leaves. *Antioxidants* **2021**, *10*, 863. https://doi.org/10.3390/antiox10060863

Received: 16 May 2021
Accepted: 26 May 2021
Published: 27 May 2021

Publisher's Note: MDPI stays neutral with regard to jurisdictional claims in published maps and institutional affiliations.

Copyright: © 2021 by the authors. Licensee MDPI, Basel, Switzerland. This article is an open access article distributed under the terms and conditions of the Creative Commons Attribution (CC BY) license (http://creativecommons.org/licenses/by/4.0/).

Content

Table S1. Reference standards used for the qualitative and quantitative analysis by HPLC-DAD-ESI-tQ-MS and HPLC-UV assays.

Table S2. Regression equations, correlation coefficients, standard deviation, limits of detection, limits of quantification and linear ranges for 55 reference standards.

Table S3. Retention times, UV- and ESI-MS spectral data of compounds **1–171** found in *Rhododendron adamsii*

Table S4. UV-spectral patterns of compounds found in *R. adamsii*.

Figure S1. High-performance anion-exchange chromatography with photodiode detection chromatograms of free carbohydrates of *Rhododendron adamsii* leaves and reference standards.

Figure S2. High-performance liquid chromatography with photodiode detection chromatograms of triterpenic acids of *R. adamsii* leaves and reference standards.

Figure S3. HPLC-PDA chromatograms of ether and methanolic extracts of fresh *R. adamsii* leaves.

Figure S4. Yield of ether extract from *R. adamsii* leaves collected in various months.

Table S1. Reference standards used for the qualitative and quantitative analysis by HPLC-DAD-ESI-tQ-MS and HPLC-UV assays.

No	Compound	Purity (≥), %	Manufacturer (cat. No)*	Used for analysis (compound No in Table 2)
**3**	Malic acid	99	Sigma (W265501)	**3**
**4**	Citric acid	99	Sigma (251275)	**4**
**5**	Tartaric acid	99	Sigma (W304401)	**5**
**6**	Succinic acid	99	Sigma (W471920)	**6**
**7**	Fumaric acid	99	Sigma (47910)	**7**
**10**	Phlorin (phloroglucinol *O*-glucoside)	90	Sigma (PHL85690)	**8**, **9**, **10**, **11**, **12**, **13**, **14**
**17**	Arbutin (hydroquinone *O*-glucoside)	98	Sigma (A4256)	**15**, **16**, **17**, **19**, **20**
**21**	Sakakin (orcinol *O*-glucoside)	90	Sigma (PHL83267)	**18**, **21**, **24**
**45**	Dihydroquercetin 3-*O*-rhamnoside	95	Sigma (PHL80356)	**42**, **43**, **44**, **45**, **102**, **109**, **112**, **113**
**50**	Myricetin 3-*O*-rutinoside	98	Wuhan (TBW01950)	**50**
**51**	Myricetin 3-*O*-galactoside	85	Sigma (SML00249)	**51**
**52**	Isomyricitrin (myricetin 3-*O*-glucoside)	99	Extrasynthese (1357S)	**36**, **37**, **46**, **47**, **49**, **56**, **91**, **92**, **93**, **94**, **97**, **99**, **106**, **107**, **108**, **111**, **118**, **119**, **165**, **166**, **167**, **168**, **169**
**53**	Rutin (quercetin 3-*O*-rutinoside)	95	Sigma (R2303)	**53**
**54**	Hyperoside (quercetin 3-*O*-galactoside)	98	Extrasynthese (1027S)	**54**
**55**	Isoquercitrin (quercetin 3-*O*-glucoside)	98	Sigma (16654)	**38**, **48**, **100**, **101**, **103**, **104**, **110**, **114**, **115**, **121**, **122**
**57**	Avicularin (quercetin 3-*O*-arabinoside)	95	Sigma (75759)	**57**
**58**	Myricitrin (myricetin 3-*O*-rhamnoside)	99	Sigma (91255)	**58**
**59**	Quercitrin (quercetin 3-*O*-rhamnoside)	98	Sigma (740580)	**59**
**60**	Juglanin (kaempferol 3-*O*-arabinoside)	95	MCE (HYN3433)	**60**
**61**	Afzelin (kaempferol 3-*O*-rhamnoside)	98	Sigma (PHL83864)	**61**
**62**	Dihydroquercetin (taxifolin)	95	Sigma (PHL89284)	**62**
**63**	Phloretin	99	Sigma (P7912)	**63**
**64**	Dihydrokaempferol (aromadendrin)	95	Sigma (SMB00175)	**64**
**65**	Myricetin	96	Sigma (70050)	**65**
**66**	Quercetin	95	Sigma (Q4951)	**66**
**67**	Luteolin	98	Sigma (L9283)	**67**
**68**	Apigenin	95	Sigma (10798)	**68**
**69**	Isorhamnetin	95	Sigma (17794)	**69**
**73**	Kaempferol	99	Extrasynthese (1124S)	**73**
**74**	Farrerol	99	MCE (HYN0344)	**74**
**86**	1-*O*-Caffeoylquinic acid	98	ChemFaces (CFN99121)	**86**
**89**	Vanillic acid 4-*O*-glucoside	98	ChemFaces (CFN95257)	**89**
**95**	5-*O*-Caffeoylquinic acid	95	Sigma (C3878)	**95**
**96**	3-*O*-Caffeoylquinic acid	98	Sigma (94419)	**96**
**98**	4-*O*-Caffeoylquinic acid	98	Sigma (65969)	**98**
**105**	Miquelianin (quercetin 3-*O*-glucuronide)	90	Sigma (90733)	**105**
**120**	Cannabigerorcinic acid	98	Cayman (23258)	**70**, **71**, **72**, **75**, **76**, **77**, **78**, **79**, **80**, **81**, **82**, **83**, **120**, **116**, **117**, **123**, **124**, **129**
**137**	Grifolic acid	98	ChemFaces (CFN97284)	**125**, **126**, **127**, **128**, **130**, **131**, **132**, **137**, **138**, **139**, **140**
**141**	Daurichromenic acid	98	ChemFaces (CFN97302)	**133**, **134**, **135**, **136**, **141**, **141**, **142**, **143**, **144**, **145**
**148**	Gallic acid	97	Sigma (G7384)	**146**, **147**, **148**, **149**, **150**, **151**
**151**	Gallic acid *O*-methyl ester	98	Sigma (274194)	**151**
**154**	Procyanidin B_1	90	Extrasynthese (0983)	**154**
**155**	Catechin	99	Extrasynthese (0976S)	**152**, **153**, **155**, **157**

Table S1. *Cont*.

No	Compound	Purity (≥), %	Manufacturer (cat. No)*	Used for analysis (compound No in Table 2)
**158**	Procyanidin B_2	90	Sigma (PHL89552)	**158**
**159**	Epicatechin	99	Extrasynthese (0977S)	**159**
**160**	Procyanidin C_1	90	Extrasynthese (0988)	**160**
**161**	Catechin 3-*O*-gallate	98	Extrasynthese (0972S)	**156**, **161**, **162**
**164**	Epicatechin 3-*O*-gallate	97.5	Extrasynthese (0978S)	**164**
**170**	Quercetin 3-*O*-(6′′-*O*-galloyl)-glucoside	98	ChemFaces (CFN91064)	**170**, **171**
	Saccharose	99	Sigma (S7903)	**1**
	Glucose	99	Sigma (49161)	**2**
	Ursolic acid	90	Sigma (216753)	**22**, **23**, **25**, **26**, **27**, **28**, **29**, **30**, **31**, **32**, **33**, **34**, **35**
	Dihydromyricetin	98	Sigma (SML0295)	**39**, **40**, **41**
	Protocatechuic acid	97	Sigma (37580)	**84**, **85**
	Vanillic acid	97	Sigma (H36001)	**88**, **89**, **90**

* Manufacturers list: Cayman—Cayman Chemicals (Ann Arbor, MI, USA); ChemFaces—ChemFaces (Wuhan, Hubei, PRC); Extrasynthese—Extrasynthese (Lyon, France); MCE—MCE Med Chem Express (Monmouth, NJ, USA); Sigma—Sigma-Aldrich (St. Louis, MO, USA); Wuhan—Wuhan Chem Norm Biotech Co., Ltd. (Wuhan, PRC).

Table S2. Regression equations, correlation coefficients (r^2), standard deviation (S_{YX}), limits of detection (LOD), limits of quantification (LOQ) and linear ranges for 55 reference standards.

Compound	Ionization [a]	CE [b] (eV)	Regression equation [c]		r^2	S_{YX}	LOD/LOQ (μg/mL)	Linear range (μg/mL)
			a	*b*·10⁶				
1-*O*-Caffeoylquinic acid	N	-15	2.5394	-1.2360	0.9994	$0.45 \cdot 10^{-2}$	0.006/0.02	0.02–300.0
3-*O*-Caffeoylquinic acid	N	-15	2.4176	-1.5647	0.9994	$0.40 \cdot 10^{-2}$	0.005/0.02	0.02–300.0
4-*O*-Caffeoylquinic acid	N	-15	2.7365	-1.0690	0.9996	$0.51 \cdot 10^{-2}$	0.006/0.02	0.02–300.0
5-*O*-Caffeoylquinic acid	N	-15	2.9021	-1.4184	0.9998	$0.39 \cdot 10^{-2}$	0.004/0.01	0.02–300.0
Afzelin (kaempferol 3-*O*-rhamnoside)	N	-20	2.0859	-0.9171	0.9980	$6.18 \cdot 10^{-2}$	0.03/0.09	0.10–100.0
Apigenin	N	-15	5.8022	-0.8040	0.9990	$1.14 \cdot 10^{-2}$	0.007/0.02	0.02–500.0
Arbutin (hydroquinone *O*-glucoside)	N	-10	0.1756	-0.0144	0.9967	$3.01 \cdot 10^{-2}$	0.56/1.71	2.00–850.0
Avicularin (quercetin 3-*O*-arabinoside)	N	-20	1.4412	-0.6211	0.9930	$11.25 \cdot 10^{-2}$	0.26/0.78	0.80–100.0
Cannabigerorcinic acid	N	-15	1.1123	-1.0289	0.9862	$0.41 \cdot 10^{-2}$	0.01/0.04	0.05–400.0
Catechin	N	-20	0.9562	-0.0521	0.9971	$7.79 \cdot 10^{-2}$	0.27/0.82	0.90–100.0
Catechin 3-*O*-gallate	N	-20	1.3387	-0.0284	0.9981	$9.50 \cdot 10^{-2}$	0.23/0.71	0.80–100.0
Citric acid	N	-10	0.9518	-0.0267	0.9990	$1.03 \cdot 10^{-2}$	0.03/0.10	0.10–100.0
Daurichromenic acid	N	-30	1.1269	-0.9560	0.9864	$0.93 \cdot 10^{-2}$	0.02/0.08	0.10–400.0
Dihydrokaempferol (aromadendrin)	N	-15	3.6748	-0.7069	0.9987	$0.90 \cdot 10^{-2}$	0.008/0.02	0.02–400.0
Dihydromyricetin	N	-15	4.9634	-0.5047	0.9972	$0.63 \cdot 10^{-2}$	0.004/0.01	0.01–400.0
Dihydroquercetin (taxifolin)	N	-15	4.1069	-0.5637	0.9989	$0.77 \cdot 10^{-2}$	0.006/0.02	0.02–400.0
Dihydroquercetin 3-*O*-rhamnoside	N	-20	2.5078	-0.6342	0.9991	$0.96 \cdot 10^{-2}$	0.01/0.03	0.04–400.0
Epicatechin	N	-20	1.0828	-0.0456	0.9973	$6.85 \cdot 10^{-2}$	0.21/0.63	0.70–100.0
Epicatechin 3-*O*-gallate	N	-20	1.5152	-0.0523	0.9979	$12.67 \cdot 10^{-2}$	0.28/0.84	0.90–100.0
Farrerol	N	-10	6.3710	-0.9634	0.9985	$0.92 \cdot 10^{-2}$	0.004/0.01	0.02–400.0
Fumaric acid	N	-10	0.8615	-0.0364	0.9982	$2.03 \cdot 10^{-2}$	0.03/0.07	0.10–100.0
Gallic acid	N	-10	2.6538	-0.1376	0.9990	$1.17 \cdot 10^{-2}$	0.01/0.04	0.10–100.0
Gallic acid *O*-methyl ester	N	-15	1.9478	-0.2369	0.9984	$1.03 \cdot 10^{-2}$	0.02/0.05	0.05–250.0
Glucose	N	-10	1.5632	-0.0376	0.9983	$5.14 \cdot 10^{-2}$	0.11/0.33	0.40–100.0
Grifolic acid	N	-25	1.0627	-1.2531	0.9893	$0.62 \cdot 10^{-2}$	0.02/0.06	0.10–400.0
Hyperoside (quercetin 3-*O*-galactoside)	N	-20	1.4689	-0.3641	0.9990	$5.69 \cdot 10^{-2}$	0.12/0.38	0.40–400.0
Isomyricitrin (myricetin 3-*O*-glucoside)	N	-20	2.6340	-0.2411	0.9973	$2.74 \cdot 10^{-2}$	0.03/0.10	0.10–350.0
Isoquercitrin (quercetin 3-*O*-glucoside)	N	-20	1.8267	-0.4160	0.9990	$11.73 \cdot 10^{-2}$	0.21/0.67	0.70–400.0
Isorhamnetin	N	-15	1.1541	-0.4691	0.9987	$1.06 \cdot 10^{-2}$	0.03/0.10	0.10–350.0
Juglanin (kaempferol 3-*O*-arabinoside)	N	-20	2.0384	-0.3640	0.9975	$2.02 \cdot 10^{-2}$	0.03/0.10	0.10–350.0
Kaempferol	N	-10	1.2416	-0.3615	0.9901	$3.02 \cdot 10^{-2}$	0.08/0.24	0.3–100.0
Luteolin	N	-15	7.064	-1.533	0.9992	$1.92 \cdot 10^{-2}$	0.009/0.03	0.03–500.0
Malic acid	N	-10	0.9911	-0.0379	0.9988	$2.05 \cdot 10^{-2}$	0.07/0.21	0.3–100.0
Miquelianin (quercetin 3-*O*-glucuronide)	N	-20	1.6705	-0.4374	0.9988	$12.79 \cdot 10^{-2}$	0.25/0.77	0.8–100.0
Myricetin	N	-15	0.9375	-0.7631	0.9982	$1.42 \cdot 10^{-2}$	0.05/0.15	0.20–350.0
Myricetin 3-*O*-galactoside	N	-20	2.4751	-0.2081	0.9976	$2.04 \cdot 10^{-2}$	0.03/0.10	0.10–350.0

Table S2. *Cont.*

Compound	Ionization [a]	CE [b] (eV)	Regression equation [c]		r^2	S_{YX}	LOD/LOQ (µg/mL)	Linear range (µg/mL)
			a	$b \cdot 10^6$				
Myricetin 3-*O*-rutinoside	N	-25	1.9634	-0.7458	0.9963	$2.59 \cdot 10^{-2}$	0.04/0.14	0.20–350.0
Myricitrin (myricetin 3-*O*-rhamnoside)	N	-20	1.8233	-0.7962	0.9975	$2.02 \cdot 10^{-2}$	0.04/0.11	0.20–350.0
Phloretin	N	-15	2.0330	-0.2516	0.9992	$1.09 \cdot 10^{-2}$	0.02/0.05	0.05–300.0
Phlorin (phloroglucinol *O*-glucoside)	N	-10	0.1537	-0.0189	0.9960	$3.83 \cdot 10^{-2}$	0.82/2.49	2.50–850.0
Procyanidin B_1	N	-30	1.3722	-0.0829	0.9973	$9.93 \cdot 10^{-2}$	0.24/0.72	0.80–100.0
Procyanidin B_2	N	-30	1.3620	-0.0820	0.9961	$9.91 \cdot 10^{-2}$	0.21/0.72	0.80–100.0
Procyanidin C_1	N	-20	1.0634	-0.0933	0.9902	$10.01 \cdot 10^{-2}$	0.31/0.94	1.00–100.0
Protocatechuic acid	N	-10	1.9610	-0.5271	0.9993	$0.94 \cdot 10^{-2}$	0.02/0.05	0.05–250.0
Quercetin	N	-15	1.1105	-0.3211	0.9937	$4.18 \cdot 10^{-2}$	0.12/0.38	0.40–400.0
Quercetin 3-*O*-(6′′-*O*-galloyl)-glucoside	N	-25	1.1492	-0.6010	0.9980	$4.68 \cdot 10^{-2}$	0.14/0.41	0.50–400.0
Quercitrin (quercetin 3-*O*-rhamnoside)	N	-20	1.9871	-0.6871	0.9984	$5.63 \cdot 10^{-2}$	0.09/0.28	0.40–400.0
Rutin (quercetin 3-*O*-rutinoside)	N	-20	1.2716	-0.7389	0.9897	$9.14 \cdot 10^{-2}$	0.23/0.72	0.80–400.0
Sucrose	N	-10	1.6278	-0.0428	0.9990	$7.11 \cdot 10^{-2}$	0.14/0.44	0.50–100.0
Sakakin (orcinol *O*-glucoside)	N	-10	0.2569	-0.0316	0.9973	$2.52 \cdot 10^{-2}$	0.33/1.00	1.00–750.0
Succinic acid	N	-10	1.4611	-0.0731	0.9982	$4.08 \cdot 10^{-2}$	0.09/0.27	0.30–100.0
Tartaric acid	N	-10	1.5330	-0.0863	0.9985	$4.15 \cdot 10^{-2}$	0.09/0.27	0.30–100.0
Ursolic acid	N	-20	2.6370	-0.5733	0.9952	$2.85 \cdot 10^{-2}$	0.03/0.11	0.20–250.0
Vanillic acid	N	-10	1.5379	-0.6220	0.9990	$0.99 \cdot 10^{-2}$	0.02/0.06	0.10–250.0
Vanillic acid 4-*O*-glucoside	N	-15	1.0864	-0.5271	0.9983	$1.04 \cdot 10^{-2}$	0.03/0.10	0.10–500.0

[a] Ionization mode : N—negative. [b] CE—collision energy. [c] Regression equation: $y = a \cdot x + b$.

Table S3. Retention times (t_R), UV- and ESI-MS spectral data of compounds **1–171** found in *Rhododendron adamsii*.

No	t_R (min) [a]	Compound [b]	UV pattern [c]	CE (eV) [d]	ESI-MS (*m*/*z*)		
					[M-H]⁻ [e]	MS/MS (*I*, %) [f]	Adduct ions
**1**	0.62 [I]	*O*-Hexosyl-hexose [L] [26]	Nil	-10	341		
**2**	0.76 [I]	Hexose [L] [26]	Nil	-10	179		
**3**	0.92 [I]	Malic acid [R] [26,29]	Nil	-10	133		
**4**	1.05 [I]	Citric acid [R] [26,29]	Nil	-10	191		
**5**	1.26 [I]	Tartaric acid [R] [26,29]	Nil	-10	149		
**6**	1.42 [I]	Succinic acid [R] [26,29]	Nil	-10	117		
**7**	1.51 [I]	Fumaric acid [R] [26,29]	Nil	-10	115		
**8**	2.48 [I]	Phloroglucinol di-*O*-hexoside [L] [44]	PGL	-10	449	[449]: 287 (12), 125 (100)	
**9**	2.67 [I]	Phloroglucinol di-*O*-hexoside [L] [44]	PGL	-10	449	[449]: 287 (12), 125 (100)	
**10**	2.75 [I]	Phlorin (phloroglucinol *O*-glucoside) [R] [44]	PGL	-10	287	[287]: 125	
**11**	2.93 [I]	Phloroglucinol di-*O*-hexoside-*O*-acetate [L] [44]	PGL	-15	491	[491]: 449 (22), 287 (10), 125 (100)	
**12**	3.09 [I]	Phloroglucinol di-*O*-hexoside-*O*-acetate [L] [44]	PGL	-15	491	[491]: 449 (18), 287 (9), 125 (100)	
**13**	3.18 [I]	Phloroglucinol di-*O*-hexoside-di-*O*-acetate [L] [44]	PGL	-15	533	[533]: 491 (5), 449 (26), 287 (12), 125 (100)	
**14**	3.24 [I]	Phloroglucinol di-*O*-hexoside-di-*O*-acetate [L] [44]	PGL	-15	533	[533]: 491 (3), 449 (20), 287 (10), 125 (100)	
**15**	3.31 [I]	Hydroquinone di-*O*-hexoside [L] [45,46]	HQU	-10	433	[433]: 271 (100), 109 (12)	
**16**	3.43 [I]	Hydroquinone di-*O*-hexoside [L] [45,46]	HQU	-10	433	[433]: 271 (100), 109 (9)	
**17**	3.64 [I]	Arbutin (hydroquinone *O*-glucoside) [R] [45,46]	HQU	-10	271	[271]: 109	
**18**	3.69 [I]	Orcinol di-*O*-hexoside [L] [47]	ORC	-10	447	[447]: 285 (36), 123 (100)	
**19**	3.81 [I]	Hydroquinone di-*O*-hexoside-*O*-methyl ester [L] [45,46]	HQU	-10	447	[447]: 285 (23), 123 (100), 109 (12)	
**20**	3.92 [I]	Hydroquinone *O*-hexoside-*O*-methyl ester [L] [45,46]	HQU	-10	285	[285]: 123 (100), 109 (22)	
**21**	4.09 [I]	Sakakin (orcinol *O*-glucoside) [R] [47]	ORC	-10	285	[285]: 123	
**22**	4.26 [I]	Ursolic acid tri-*O*-hexoside [L] [33,48]	Nil	-20	941	[941]: 779 (10), 617 (100), 455 (54)	
**23**	4.31 [I]	Ursolic acid tri-*O*-hexoside [L] [33,48]	Nil	-20	941	[941]: 779 (14), 617 (100), 455 (43)	
**24**	4.47 [I]	Orcinol *O*-hexoside-*O*-acetate [L] [47]	ORC	-15	327	[327]: 285 (16), 123 (100)	

Table S3. *Cont.*

No	t_R (min) [a]	Compound [b]	UV pattern [c]	CE (eV) [d]	ESI-MS (*m*/*z*) [M-H]⁻ [e]	MS/MS (*I*, %) [f]	Adduct ions
**25**	4.58 [I]	Ursolic acid di-*O*-hexoside [L] [33,48]	Nil	-20	779	[779]: 617 (100), 455 (37)	
**26**	4.74 [I]	Ursolic acid di-*O*-hexoside [L] [33,48]	Nil	-20	779	[779]: 617 (100), 455 (34)	
**27**	4.79 [I]	Ursolic acid tri-*O*-hexoside-*O*-acetate [L] [33,48]	Nil	-20	983	[983]: 941 (26), 779 (8), 617 (100), 455 (43)	
**28**	4.97 [I]	Ursolic acid tri-*O*-hexoside-*O*-acetate [L] [33,48]	Nil	-20	983	[983]: 941 (20), 779 (4), 617 (100), 455 (35)	
**29**	5.18 [I]	Ursolic acid di-*O*-hexoside-*O*-acetate [L] [33,48]	Nil	-20	821	[821]: 779 (18), 617 (100), 455 (29)	
**30**	5.46 [I]	Ursolic acid *O*-hexoside [L] [33,48]	Nil	-20	617	[617]: 455	
**31**	5.53 [I]	Ursolic acid di-*O*-hexoside-di-*O*-acetate [L] [33,48]	Nil	-20	863	[863]: 821 (9), 779 (22), 659 (8), 617 (100), 455 (31)	
**32**	5.74 [I]	Ursolic acid di-*O*-hexoside-di-*O*-acetate [L] [33,48]	Nil	-20	863	[863]: 821 (11), 779 (25), 659 (4), 617 (100), 455 (25)	
**33**	7.87 [I]	Ursolic acid *O*-hexoside-*O*-acetate [L] [33,48]	Nil	-20	659	[659]: 617 (100), 455 (30)	
**34**	8.28 [I]	Ursolic acid *O*-hexoside-*O*-acetate [L] [33,48]	Nil	-20	659	[659]: 617 (100), 455 (35)	
**35**	10.26 [I]	Ursolic acid *O*-hexoside-di-*O*-acetate [L] [33,48]	Nil	-20	701	[701]: 659 (3), 617 (100), 455 (29)	
**36**	2.72 [II]	Myricetin tri-*O*-hexoside-tri-*O*-desoxyhexoside [L] [49–51]	MYR	-30	1241	[1241]: 1095 (100), 949 (22), 933 (2)<br>[949]: 803 (100), 641 (36), 479 (7)<br>[479]: 317	
**37**	2.79 [II]	Myricetin tri-*O*-hexoside-di-*O*-desoxyhexoside [L] [49–51]	MYR	-30	1095	[1095]: 949 (100), 803 (27), 641 (9)<br>[803]: 641 (100), 479 (31)<br>[479]: 317	
**38**	2.97 [II]	Quercetin tri-*O*-hexoside-di-*O*-desoxyhexoside [L] [12,13]	QUE	-25	1079	[1079]: 933 (100), 787 (21), 625 (3)<br>[787]: 625 (100), 463 (23)<br>[463]: 301	
**39**	3.22 [II]	Dihydromyricetin di-*O*-hexoside [L] [12,52]	DHM	-20	643	[643]: 481 (100), 319 (27)	
**40**	3.45 [II]	Dihydromyricetin *O*-hexoside [L] [12,52]	DHM	-20	481	[481]: 319	
**41**	3.52 [II]	Dihydroquercetin di-*O*-hexoside-di-*O*-desoxyhexoside [L] [12,52]	DHQ	-25	919	[919]: 773 (12), 627 (100), 611 (35)<br>[627]: 465 (100), 303 (5)	

Table S3. *Cont*.

No	t_R (min) [a]	Compound [b]	UV pattern [c]	CE (eV) [d]	ESI-MS (*m*/*z*)		
					[M-H]⁻ [e]	MS/MS (*I*, %) [f]	Adduct ions
**42**	3.64 [II]	Dihydroquercetin di-*O*-hexoside-*O*-desoxyhexoside [L] [12,52]	DHQ	-25	773	[773]: 627 (100), 465 (32)<br>[465]: 303	
**43**	3.72 [II]	Dihydroquercetin *O*-hexoside-*O*-desoxyhexoside [L] [12,52]	DHQ	-25	611	[611]: 465 (100), 303 (28)	
**44**	3.78 [II]	Dihydroquercetin *O*-hexoside [L] [12,52]	DHQ	-20	465	[465]: 303	
**45**	3.83 [II]	Dihydroquercetin 3-*O*-rhamnoside [R] [12,52]	DHQ	-20	449	[449]: 303	
**46**	4.09 [II]	Myricetin di-*O*-hexoside-di-*O*-desoxyhexoside [L] [49–51]	MYR	-25	933	[933]: 787 (25), 641 (100)<br>[641]: 479 (100), 317 (27)	
**47**	4.18 [II]	Myricetin di-*O*-hexoside-*O*-desoxyhexoside [L] [49–51]	MYR	-25	787	[787]: 641 (100), 479 (35)<br>[479]: 317	
**48**	4.26 [II]	Quercetin di-*O*-hexoside-*O*-desoxyhexoside [L] [12,13]	QUE	-25	771	[771]: 625 (21), 463 (100), 301 (12)	
**49**	4.31 [II]	Myricetin *O*-hexoside-*O*-desoxyhexoside [L] [49–51]	MYR	-25	625	[625]: 479 (100), 317 (25)	
**50**	4.43 [II]	Myricetin 3-*O*-rutinoside [R] [49–51]	MYR	-25	625	[625]: 479 (100), 317 (3)	
**51**	4.51 [II]	Myricetin 3-*O*-galactoside [R] [49–51]	MYR	-20	479	[479]: 317	
**52**	4.58 [II]	Isomyricitrin (myricetin 3-*O*-glucoside) [R] [49–51]	MYR	-20	479	[479]: 317	
**53**	4.74 [II]	Rutin (quercetin 3-*O*-rutinoside) [R] [12,13]	QUE	-20	609	[609]: 463 (100), 301 (15)	
**54**	4.78 [II]	Hyperoside (quercetin 3-*O*-galactoside) [R] [12,13]	QUE	-20	463	[463]: 301	
**55**	4.83 [II]	Isoquercitrin (quercetin 3-*O*-glucoside) [R] [12,13]	QUE	-20	463	[463]: 301	
**56**	4.97 [II]	Myricetin *O*-pentoside [L] [49–51]	MYR	-20	449	[449]: 317	
**57**	5.11 [II]	Avicularin (quercetin 3-*O*-arabinoside) [R] [12,13]	QUE	-20	433	[433]: 301	
**58**	5.26 [II]	Myricitrin (myricetin 3-*O*-rhamnoside) [R] [49–51]	MYR	-20	463	[463]: 317	
**59**	5.63 [II]	Quercitrin (quercetin 3-*O*-rhamnoside) [R] [12,13]	QUE	-20	433	[433]: 301	
**60**	5.77 [II]	Juglanin (kaempferol 3-*O*-arabinoside) [R] [35]	KAE	-20	417	[417]: 285	
**61**	6.29 [II]	Afzelin (kaempferol 3-*O*-rhamnoside) [R] [35]	KAE	-20	431	[431]: 285	
**62**	8.25 [II]	Dihydroquercetin (taxifloin) [R] [12,52]	DHQ	-15	303		
**63**	8.68 [II]	Phloretin [R] [35]	PHL	-15	273		

Table S3. *Cont.*

No	t_R (min) [a]	Compound [b]	UV pattern [c]	CE (eV) [d]	ESI-MS (*m/z*)		
					[M-H]⁻ [e]	MS/MS (*I*, %) [f]	Adduct ions
**64**	8.83 [II]	Dihydrokaempferol (aromadendrin) [R] [12,52]	DHK	-15	287		
**65**	9.01 [II]	Myricetin [R] [49–51]	MYR	-15	317		
**66**	9.23 [II]	Quercetin [R] [12,13]	QUE	-15	301		
**67**	9.27 [II]	Luteolin [R] [35]	LUT	-15	285		
**68**	9.37 [II]	Apigenin [R] [35]	API	-15	269		
**69**	9.51 [II]	Isorhamnetin [R] [35]	QUE	-15	315		
**70**	9.76 [II]	Cannabigerorcinic acid *O*-methyl ester di-*O*-hexoside [L] [53,54]	CGA	-15	641	[641]: 479 (26), 317 (100), 303 (5)<br>[317]: 303 (28), 285 (100)<br>[303]: 285 (18), 275 (43), 234 (100), 166 (18)	
**71**	9.93 [II]	Cannabigerorcinic acid *O*-methyl ester di-*O*-hexoside [L] [53,54]	CGA	-15	641	[641]: 479 (23), 317 (100), 303 (7)<br>[317]: 303 (24), 285 (100)<br>[303]: 285 (16), 275 (51), 234 (100), 166 (22)	
**72**	10.04 [II]	Cannabigerorcinic acid *O*-methyl ester *O*-hexoside-*O*-desoxyhexoside [L] [53,54]	CGA	-15	625	[625]: 479 (31), 317 (100)<br>[317]: 303 (25), 285 (100)<br>[303]: 285 (18), 275 (45), 234 (100), 166 (26)	
**73**	10.24 [II]	Kaempferol [R] [35]	KAE	-10	285		
**74**	10.48 [II]	Farrerol [R] [35]	FAR	-10	299		
**75**	10.63 [II]	Cannabigerorcinic acid methyl ester *O*-hexoside [L] [53,54]	CGA	-20	479	[479]: 317 (42), 303 (100), 285 (93)<br>[303]: 285 (15), 275 (38), 234 (100), 166 (16)	
**76**	10.97 [II]	Cannabigerorcinic acid di-*O*-methyl ester *O*-hexoside [L] [53,54]	CGA	-22	493	[493]: 331 (19), 317 (5), 303 (100), 285 (14)<br>[303]: 285 (22), 275 (40), 234 (100), 166 (19)	
**77**	11.05 [II]	Cannabigerorcinic acid di-*O*-methyl ester *O*-hexoside [L] [53,54]	CGA	-22	493	[493]: 331 (22), 317 (7), 303 (100), 285 (11)<br>[303]: 285 (26), 275 (42), 234 (100), 166 (20)	
**78**	11.22 [II]	Cannabigerorcinic acid di-*O*-methyl ester *O*-hexoside [L] [53,54]	CGA	-22	493	[493]: 331 (21), 317 (5), 303 (100), 285 (14)<br>[303]: 285 (31), 275 (49), 234 (100), 166 (25)	
**79**	11.47 [II]	Cannabigerorcinic acid *O*-methyl ester [L] [53,54]	CGA	-30	317	[317]: 303 (27), 285 (100)<br>[303]: 285 (21), 275 (43), 234 (100), 166 (22)	635 [2M-H]
**80**	11.52 [II]	Cannabigerorcinic acid *O*-methyl ester [L] [53,54]	CGA	-30	317	[317]: 303 (26), 285 (100)<br>[303]: 285 (22), 275 (46), 234 (100), 166 (27)	
**81**	11.74 [II]	Cannabigerorcinic acid di-*O*-methyl ester [L] [53,54]	CGA	-30	331	[331]: 317 (10), 303 (100), 285 (73)<br>[303]: 285 (24), 275 (47), 234 (100), 166 (23)	
**82**	12.04 [II]	Cannabigerorcinic acid di-*O*-methyl ester [L] [53,54]	CGA	-30	331	[331]: 317 (9), 303 (100), 285 (75)<br>[303]: 285 (27), 275 (40), 234 (100), 166 (18)	

Table S3. *Cont.*

No	t_R (min) [a]	Compound [b]	UV pattern [c]	CE (eV) [d]	ESI-MS (*m/z*) [M-H]⁻ [e]	MS/MS (*I*, %) [f]	Adduct ions
**83**	13.15 [II]	Cannabigerorcinic acid tri-*O*-methyl ester [L] [53,54]	CGA	-30	345	[345]: 331 (5), 317 (63), 303 (100), 285 (52)<br>[303]: 285 (24), 275 (49), 234 (100), 166 (16)	
**84**	0.68 [III]	Protocatechuic acid di-*O*-hexoside [L] [35]	PCA	-15	477	[477]: 315 (22), 153 (100)	
**85**	0.89 [III]	Protocatechuic acid *O*-hexoside [L] [35]	PCA	-15	315	[315]: 153	
**86**	2.42 [III]	1-*O*-Caffeoylquinic acid [R] [55]	CQA	-15	353	[353]: 191 (100), 179 (15), 173 (3), 135 (5)	
**87**	2.71 [III]	Vanillic/isovanillic acid *O*-hexoside [L] [35]	VA	-15	329	[329]: 167	
**88**	2.76 [III]	Vanillic/isovanillic acid *O*-hexoside [L] [35]	VA	-15	329	[329]: 167	
**89**	2.81 [III]	Vanillic acid 4-*O*-glucoside [R] [35]	VA	-15	329	[329]: 167	
**90**	2.95 [III]	Vanillic/isovanillic acid *O*-hexoside [L] [35]	VA	-15	329	[329]: 167	
**91**	3.42 [III]	Myricetin di-*O*-hexoside-di-*O*-hexuronide [L] [49–51]	MYR	-30	993	[993]: 817 (82), 655 (100)<br>[655]: 479 (100), 317 (23)	
**92**	3.54 [III]	Myricetin di-*O*-hexoside-di-*O*-hexuronide [L] [49–51]	MYR	-30	993	[993]: 817 (89), 655 (100)<br>[655]: 479 (100), 317 (21)	
**93**	3.74 [III]	Myricetin di-*O*-hexoside-*O*-hexuronide [L] [49–51]	MYR	-25	817	[817]: 641 (100), 479 (35)<br>[479]: 317	
**94**	4.11 [III]	Myricetin *O*-hexoside-di-*O*-hexuronide [L] [49–51]	MYR	-25	831	[831]: 655 (12), 479 (100), 317 (20)	
**95**	4.26 [III]	5-*O*-Caffeoylquinic acid [R] [55]	CQA	-15	353	[353]: 191 (100), 179 (4), 165 (15)	
**96**	4.51 [III]	3-*O*-Caffeoylquinic acid [R] [55]	CQA	-15	353	[353]: 191 (100), 179 (81), 135 (22)	
**97**	4.67 [III]	Myricetin *O*-hexoside-*O*-hexuronide [L] [49–51]	MYR	-25	655	[655]: 479 (100), 317 (25)	
**98**	4.72 [III]	4-*O*-Caffeoylquinic acid [R] [55]	CQA	-15	353	[353]: 191 (19), 179 (100), 173 (91), 135 (12)	
**99**	4.89 [III]	Myricetin *O*-hexuronide [L] [49–51]	MYR		493	[493]: 317	
**100**	5.03 [III]	Quercetin di-*O*-hexoside-*O*-hexuronide [L] [12,13]	QUE	-25	801	[801]: 625 (11), 463 (100), 301 (14)	
**101**	5.14 [III]	Quercetin *O*-hexoside-di-*O*-hexuronide [L] [12,13]	QUE	-25	815	[801]: 639 (5), 463 (100), 301 (12)	
**102**	5.26 [III]	Dihydroquercetin *O*-hexuronide [L] [12,51]	DHQ	-25	479	[479]: 303	

Table S3. *Cont.*

No	t_R (min) [a]	Compound [b]	UV pattern [c]	CE (eV) [d]	ESI-MS (*m/z*)		
					[M-H]⁻ [e]	MS/MS (*I*, %) [f]	Adduct ions
**103**	5.38 [III]	Quercetin *O*-hexoside-*O*-hexuronide [L] [12,13]	QUE	-25	639	[639]: 463 (100), 301 (10)	
**104**	5.63 [III]	Quercetin *O*-hexoside-*O*-hexuronide [L] [12,13]	QUE	-25	639	[639]: 463 (100), 301 (15)	
**105**	5.69 [III]	Miquelianin (quercetin 3-*O*-glucuronide) [R] [12,13]	QUE	-20	477	[477]: 301	
**106**	6.01 [III]	Myricetin *O*-hexuronide-*O*-acetate [L] [48–50]	MYR	-30	535	[535]: 493 (100), 317 (28)	
**107**	6.78 [III]	Myricetin *O*-hexoside-di-*O*-acetate [L] [49–51]	MYR	-30	563	[563]: 521 (25), 479 (100), 317 (33)	
**108**	6.92 [III]	Myricetin *O*-hexouronide-di-*O*-acetate [L] [49–51]	MYR	-30	577	[577]: 535 (3), 493 (100), 317 (18)	
**109**	7.22 [III]	Dihydroquercetin *O*-hexuronide-*O*-acetate [L]	DGQ	-25	521	[521]: 479 (100), 303 (9)	
**110**	7.36 [III]	Quercetin *O*-hexuronide-*O*-acetate [L]	QUE	-25	519	[519]: 477 (100), 301 (12)	
**111**	7.92 [III]	Myricetin *O*-hexoside-di-*O*-acetate [L] [49–51]	MYR	-30	563	[563]: 521 (21), 479 (100), 317 (41)	
**112**	8.14 [III]	Dihydroquercetin *O*-hexuronide-di-*O*-acetate [L] [12,52]	DHQ	-25	563	[563]: 521 (7), 479 (100), 303 (9)	
**113**	8.26 [III]	Dihydroquercetin *O*-hexuronide-di-*O*-acetate [L] [12,52]	DHQ	-25	563	[563]: 521 (5), 479 (100), 303 (5)	
**114**	8.47 [III]	Quercetin *O*-hexuronide-di-*O*-acetate [L] [12,13]	QUE	-25	561	[561]: 519 (12), 477 (100), 301 (14)	
**115**	8.69 [III]	Quercetin *O*-hexuronide-di-*O*-acetate [L] [12,13]	QUE	-25	561	[561]: 519 (10), 477 (100), 301 (18)	
**116**	8.92 [III]	Cannabigerorcinic acid di-*O*-hexoside [L] [53,54]	CGA	-25	627	[627]: 465 (14), 303 (100), 285 (57)<br>[303]: 285 (25), 275 (42), 234 (100), 166 (20)	
**117**	9.01 [III]	Cannabigerorcinic acid *O*-hexoside [L] [53,54]	CGA	-25	465	[465]: 303 (73), 285 (100)<br>[303]: 285 (22), 275 (43), 234 (100), 166 (25)	
**118**	9.18 [III]	Myricetin *O*-hexoside-tri-*O*-acetate [L] [49–51]	MYR	-25	605	[605]: 563 (5), 479 (100), 317 (25)	
**119**	9.24 [III]	Myricetin *O*-hexoside-tri-*O*-acetate [L] [49–51]	MYR	-25	605	[605]: 563 (2), 479 (100), 317 (31)	

Table S3. *Cont.*

No	t_R (min) [a]	Compound [b]	UV pattern [c]	CE (eV) [d]	ESI-MS (*m*/*z*)		
					[M-H]⁻ [e]	MS/MS (*I*, %) [f]	Adduct ions
**120**	9.33 [III]	Cannabigerorcinic acid [R]	CGA	-20	303	[303]: 285 (37), 275 (50), 234 (100), 166 (25)	607 [2M-H]
**121**	9.46 [III]	Quercetin *O*-hexoside-tri-*O*-acetate [L] [12,13]	QUE	-25	589	[589]: 547 (2), 505 (14), 463 (100), 301 (11)	
**122**	9.53 [III]	Quercetin *O*-hexoside-tri-*O*-acetate [L] [12,13]	QUE	-25	589	[589]: 547 (1), 505 (10), 463 (100), 301 (18)	
**123**	9.72 [III]	Cannabigerorcinic acid *O*-acetate [L]	CGA	-20	345	[345]: 303 (82), 285 (100)<br>[303]: 285 (31), 275 (43), 234 (100), 166 (22)	
**124**	9.81 [III]	Cannabigerorcinic acid di-*O*-acetate [L]	CGA	-20	387	[387]: 345 (18), 303 (100), 285 (53)<br>[303]: 285 (29), 275 (42), 234 (100), 166 (18)	
**125**	9.93 [III]	Hydroxy-grifolic acid di-*O*-hexoside [L] [53]	GRA	-20	711	[711]: 549 (18), 387 (100), 369 (73)<br>[387]: 369 (27), 318 (53), 250 (100), 196 (18)	
**126**	9.98 [III]	Hydroxy-grifolic acid *O*-hexoside [L] [53]	GRA	-20	549	[549]: 387 (28), 369 (100)<br>[387]: 369 (25), 318 (50), 250 (100), 196 (14)	
**127**	10.09 [III]	Hydroxy-grifolic acid *O*-hexoside [L] [53]	GRA	-20	549	[549]: 387 (31), 369 (100)<br>[387]: 369 (29), 318 (48), 250 (100), 196 (16)	
**128**	10.27 [III]	Hydroxy-grifolic acid *O*-pentoside [L] [53]	GRA	-20	519	[519]: 387 (25), 369 (100)<br>[387]: 369 (25), 318 (50), 250 (100), 196 (18)	
**129**	10.40 [III]	Cannabigerorcinic acid tri-*O*-acetate [L] [53,54]	CGA	-20	429	[429]: 387 (3), 345 (12), 303 (100)<br>[303]: 285 (35), 275 (52), 234 (100), 166 (21)	
**130**	10.67 [III]	Hydroxy-grifolic acid [L] [53]	GRA	-20	387	[387]: 369 (27), 318 (53), 250 (100), 196 (22)	
**131**	10.75 [III]	Grifolic acid di-*O*-hexoside [L] [53]	GRA	-20	695	[695]: 533 (23), 371 (100), 353 (83)<br>[371]: 353 (10), 302 (63), 234 (100), 180 (5)	
**132**	10.86 [III]	Grifolic acid *O*-hexoside [L] [53]	GRA	-20	533	[533]: 371 (51), 353 (100)<br>[371]: 353 (14), 302 (61), 234 (100), 180 (9)	
**133**	10.86 [III]	Daurichromenic acid di-*O*-hexoside [L] [53,54]	DCA	-25	693	[693]: 531 (52), 369 (100)<br>[369]: 351 (12), 300 (100), 232 (31)	
**134**	11.06 [III]	Daurichromenic acid *O*-hexoside [L] [53,54]	DCA	-25	531	[531]: 369<br>[369]: 351 (10), 300 (100), 232 (29)	
**135**	11.26 [III]	Hydroxy-daurichromenic acid [L] [53,54]	HDCA	-30	385	[385]: 367 (22), 316 (100), 248 (39)	769 [2M-H]
**136**	11.43 [III]	Hydroxy-daurichromenic acid *O*-methyl ester [L] [53,54]	HDCA	-30	399	[399]: 385 (15), 367 (100)<br>[385]: 367 (25), 316 (100), 248 (42)	
**137**	11.64 [III]	Grifolic acid [R] [53]	GRA	-25	371	[371]: 353 (15), 327 (33), 302 (62), 234 (100), 180 (12)	741 [2M-H]

Table S3. *Cont.*

No	t_R (min) [a]	Compound [b]	UV pattern [c]	CE (eV) [d]	ESI-MS (*m/z*) [M-H]⁻ [e]	MS/MS (*I*, %) [f]	Adduct ions
**138**	12.72 [III]	Grifolic acid *O*-methyl ester [L] [53]	GRA	-25	385	[385]: 371 (23), 353 (100)<br>[371]: 353 (14), 302 (59), 234 (100), 180 (10)	
**139**	12.81 [III]	Grifolic acid di-*O*-methyl ester [L] [53]	GRA	-25	399	[399]: 385 (26), 367 (100)<br>[385]: 371 (20), 353 (100)<br>[371]: 353 (16), 302 (58), 234 (100), 180 (12)	
**140**	12.92 [III]	Grifolic acid *O*-methyl ester-*O*-acetate [L] [53]	GRA	-25	427	[427]: 385 (62), 371 (21), 353 (100)<br>[371]: 353 (18), 302 (60), 234 (100), 180 (14)	
**141**	13.14 [III]	Daurichromenic acid [R] [53,54]	DCA	-30	369	[369]: 351 (12), 300 (100), 232 (35)	737 [2M-H]
**142**	13.42 [III]	Daurichromenic acid *O*-acetate [L] [53,54]	DCA	-32	411	[411]: 369 (100), 351 (18)<br>[369]: 351 (14), 300 (100), 232 (32)	
**143**	13.58 [III]	Daurichromenic acid *O*-methyl ester [L] [53,54]	DCA	-35	383	[383]: 369 (25), 351 (100)<br>[369]: 351 (12), 300 (100), 232 (30)	
**144**	13.74 [III]	Daurichromenic acid *O*-methyl ester-*O*-acetate [L] [53,54]	DCA	-35	425	[425]: 383 (35), 369 (53), 351 (100)<br>[369]: 351 (14), 300 (100), 232 (31)	
**145**	14.23 [III]	Daurichromenic acid di-*O*-methyl ester [L] [53,54]	DCA	-35	397	[397]: 383 (5), 369 (63), 351 (100)<br>[369]: 351 (9), 300 (100), 232 (28)	
**146**	2.70 [IV]	Gallic acid di-*O*-hexoside [L] [28]	GA	-10	493	[493]: 331 (52), 169 (100)<br>[169]: 151 (100), 141 (10)	
**147**	3.27 [IV]	Gallic acid *O*-hexoside [L] [28]	GA	-10	331	[331]: 169<br>[169]: 151 (100), 141 (14)	
**148**	3.94 [IV]	Gallic acid [R] [28]	GA	-10	169	[169]: 151 (100), 141 (12)	
**149**	4.32 [IV]	Gallic acid *O*-methyl ester *O*-hexoside [L] [28]	GA	-15	345	[345]: 183 (100), 169 (24)<br>[169]: 151 (100), 141 (9)	
**150**	4.91 [IV]	Gallic acid *O*-methyl ester *O*-hexoside [L] [28]	GA	-15	345	[345]: 183 (100), 169 (22)<br>[169]: 151 (100), 141 (7)	
**151**	5.60 [IV]	Gallic acid *O*-methyl ester [R] [28]	GA	-15	183	[183]: 169<br>[169]: 151 (100), 141 (11)	
**152**	7.82 [IV]	Catechin/epicatechin di-*O*-hexoside [L] [28]	CAT	-20	613	[613]: 451 (53), 289 (100)	
**153**	8.51 [IV]	Catechin/epicatechin *O*-hexoside [L] [28]	CAT	-20	451	[451]: 289	
**154**	9.03 [IV]	Procyanidin B_1 [R] [28]	CAT	-30	577	[577]: 289	
**155**	9.82 [IV]	Catechin [R] [28]	CAT	-20	289	[289]: 247 (100), 191 (28), 123 (9)	
**156**	10.76 [IV]	Catechin/epicatechin *O*-hexoside-*O*-gallate [L] [28]	CAT	-20	603	[603]: 451 (33), 289 (100)	
**157**	12.15 [IV]	Catechin/epicatechin *O*-hexoside [L] [28]	CAT	-20	451	[451]: 289	
**158**	13.02 [IV]	Procyanidin B_2 [R] [28]	CAT	-30	577	[577]: 289	
**159**	13.54 [IV]	Epicatechin [R] [28]	CAT	-20	289	[289]: 247 (14), 191 (58), 123 (100)	
**160**	15.51 [IV]	Procyanidin C_1 [R] [28]	CAT	-20	865	[865]: 577 (82), 289 (100)	
**161**	19.67 [IV]	Catechin 3-*O*-gallate [R] [28]	CAT	-20	441	[441]: 289 (9), 125 (15), 109 (100)	
**162**	20.52 [IV]	Catechin/epicatechin dimer *O*-gallate [L] [28]	CAT	-30	729	[729]: 577 (100), 289 (53)	

Table S3. *Cont.*

No	t_R (min) [a]	Compound [b]	UV pattern [c]	CE (eV) [d]	ESI-MS (*m/z*)		
					[M-H]- [e]	MS/MS (*I*, %) [f]	Adduct ions
**163**	21.48 [IV]	Catechin/epicatechin dimer di-*O*-gallate [L] [28]	CAT	-30	881	[729]: 577 (100), 289 (44)	
**164**	22.26 [IV]	Epicatechin 3-*O*-gallate [R] [28]	CAT	-20	441	[441]: 289 (15), 125 (42), 109 (100)	
**165**	22.97 [IV]	Myricetin tri-*O*-hexoside-di-*O*-gallate [L] [49–51]	MYR	-30	1107	[1107]: 955 (14), 803 (83), 641 (100)<br>[641]: 479 (100), 317 (27)	
**166**	23.67 [IV]	Myricetin di-*O*-hexoside-di-*O*-gallate [L] [49–51]	MYR	-30	945	[945]: 793 (41), 641 (100)<br>[641]: 479 (100), 317 (25)	
**167**	24.43 [IV]	Myricetin di-*O*-hexoside-*O*-gallate [L] [49–51]	MYR	-30	793	[793]: 641 (14), 479 (100), 317 (21)	
**168**	25.63 [IV]	Myricetin *O*-hexoside-*O*-gallate [L] [49–51]	MYR	-25	631	[631]: 479 (100), 317 (25)	
**169**	25.83 [IV]	Myricetin *O*-hexoside-*O*-gallate [L] [49–51]	MYR	-25	631	[631]: 479 (100), 317 (30)	
**170**	26.74 [IV]	Quercetin 3-*O*-(6′′-*O*-galloyl)-glucoside [R] [12,13]	QUE	-25	615	[615]: 463 (100), 301 (12)	
**171**	28.02 [IV]	Quercetin *O*-hexoside-di-*O*-gallate [L] [12,13]	QUE	-25	767	[767]: 615 (8), 463 (100), 301 (15)	

[a] Chromatographic conditions: I—mode 1; II—mode 2; III—mode 3; IV—mode 4. [b] Compound identification was based on comparison of retention time, UV and MS spectral data with reference standard ([R]) or interpretation of UV and MS spectral data and comparison with literature data ([L]). [c] UV-patterns as listed in Table S4. [d] CE—collision energy. [e] Mass spectrometric data: deprotonated ion [M–H]-. [f] Signal intensity (percentage).

Table S4. UV-spectral patterns of compounds found in *R. adamsii*.

Name of UV-pattern	Group of compound	λ_{max}, nm
API	Apigenin	270, 335
CAT	Catechins	275 (±1)
CGA	Cannabigerorcinic acid (CGA) / CGA derivarives	220 (±2), 270 (±2), 305 (±3)
CQA	Caffeoylquinic acids	290 (±2) sh, 324 (±2)
DCA	Daurichromenic acid (DCA) / DCA derivatives	260 (±2)
DHQ	Dihydroquercetin (DHQ) / DHQ glycosides	290 (±2), 325 (±2) sh
DHK	Dihydrokaempferol (DHK) / DHK glycosides	291 (±2) sh, 330 (±2)
DHM	Dihydromyricetin (DHM) / DHM glycosides	295 (±2) sh, 320 (±4)
FAR	Farrerol	260, 330
GA	Gallic acid (GA) / GA derivatives	271 (±2)
GRA	Grifolic acid (GRA) / GRA derivatives	220 (±2), 270 (±2), 310 (±3)
HDCA	Hydroxy-daurichromenic acid (HDCA) / HDCA derivatives	230 (±3), 260 (±2)
HQU	Hydroquinone (HQU) / HQU derivatives	282 (±1)
KAE	Kaempferol (Ka) / Ka glycosides	265 (±3), 360 (±5)
LUT	Luteolin	255 sh, 265, 355
MYR	Myricetin (My) / My glycosides	255 (±3), 370 (±5)
Nil	No absorption > 200 nm	-
ORC	Orcinol (ORC) / ORC derivatives	230 (±2), 275 (±2), 280 (±3)
QUE	Quercetin (Qu) / Qu glycosides	256 (±3), 267 (±2), 362 (±4)
PCA	Protocatechuic acid glycosides	260 (±1), 291 (±2)
PGL	Phloroglucinol (PGL) / PGL derivatives	266 (±2)
PHL	Phloretin	220, 260
VA	Vanillic/isovanillic acid (VA) / VA derivatives	260 (±1), 292 (±2)

sh—shoulder.

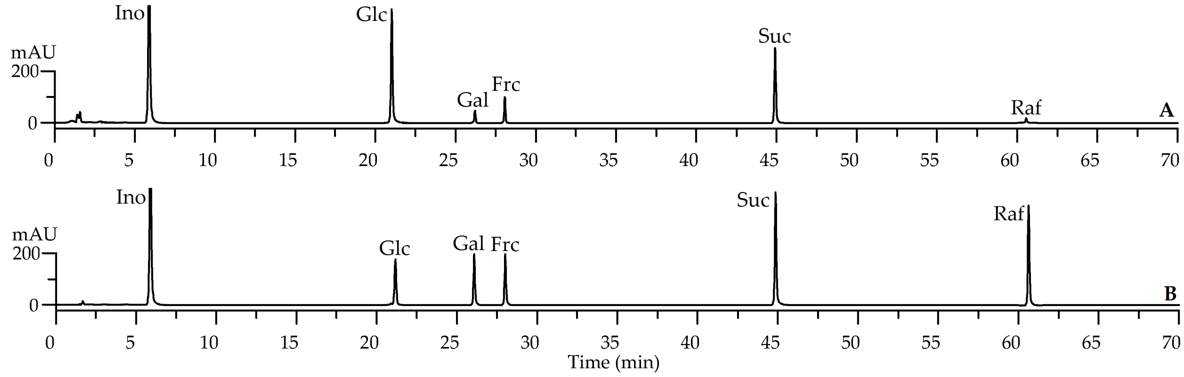

Figure S1. High-performance anion-exchange chromatography with photodiode detection (HPAEC-PDA) chromatograms of free carbohydrates of *Rhododendron adamsii* leaves (July sample, **A**) and reference standards (**B**). Chromatographic conditions: liquid chromatograph LC-20 Prominence coupled with photodiode array detector SPD-M30A (all Shimadzu, Columbia, MD, USA) and column Dionex CarboPac MA1 (4 × 250 mm, 8.5 μm; Thermo Fisher Scientific, Sunnyvale, CA, USA); isocratic elution; eluent 0.6 M NaOH; flow rate: 400 μL/min; column temperature 25 °C; sample volume 5 μL; detection wavelength 190 nm. Inositol (Ino) was used as an internal standard. Carbohydrates were signed as Frc—fructose, Gal—galactose, Glc—glucose, Raf—raffinose, and Suc—sucrose.

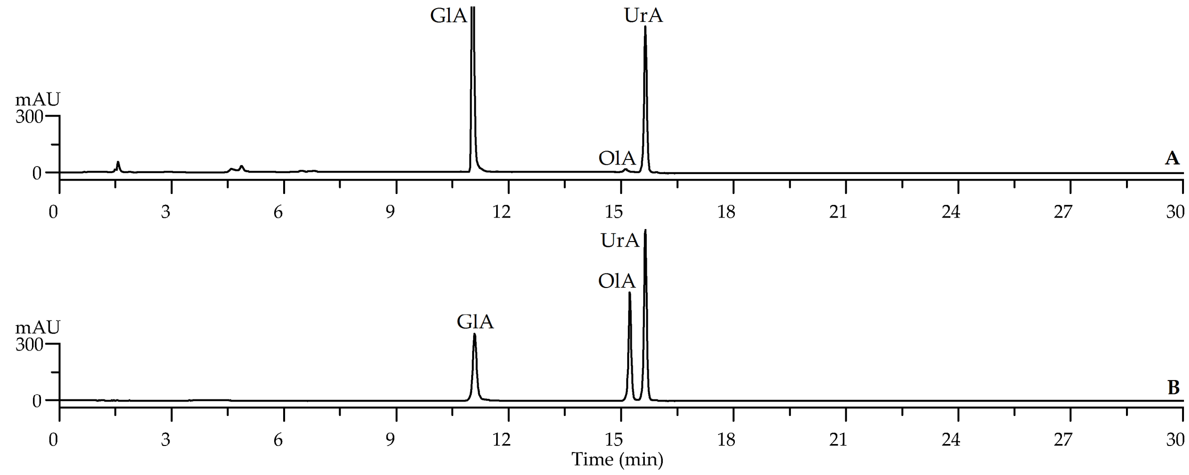

Figure S2. High-performance liquid chromatography with photodiode detection (HPLC-PDA) chromatograms of triterpenic acids of *R. adamsii* leaves (July sample, **A**) and reference standards (**B**). Sample preparation: The extract of *R. adamsii* (100 mg) was dissolved in 90% methanol (1 mL) followed by dilution of 20% methanol (8 mL), then centrifuged (6000 *g*, 15 min), and the supernatant was transferred in the volumetric flask (10 mL). Aliquot of internal standard (glycyrrhetinic acid, 200 µL; 1000 µg/mL in methanol) was added to a volumetric flask and the final volume reached 10 mL with 25% methanol in the volumetric flask (solution A). Solution A (2 mL) was passed through polyamide SPE-cartridge and eluted with water (40 mL; solution B). Solution B was concentrate in a vacuum to dryness and 5 mL of 20% HCl in acetone was added. The mixture was boiled under a reflux 60 min and after cooling it was diluted to 20 mL and transferred into a separating funnel. Liquid-liquid extraction with ethyl acetate (3 × 20 mL) was used to separate triterpenic acid aglycones. The combined organic phase was concentrated in a vacuum and the dry residue was dissolved in 5 mL of methanol. The methanolic solution was passed through Al_2O_3 SPE-cartridge, eluted with methanol (50 mL) followed with solvent removal in a vacuum, dissolving in 5 mL of methanol and HPLC-PAD analysis. Chromatographic conditions: liquid chromatograph LC-20 Prominence coupled with photodiode array detector SPD-M30A (all Shimadzu, Columbia, MD, USA) and column Acclaim™ C30 (4.6 × 250 mm, 5 µm; Thermo Fisher Scientific, Sunnyvale, CA, USA); gradient elution; eluent A 1% CH3COONH3 in water, eluent B MeCN-MeOH 3:1; flow rate: 1000 µL/min; column temperature 30 °C; sample volume 4 µL; detection wavelength 190 nm. Glycyrrhetinic acid (GlA) was used as an internal standard. Triterpenic acids were signed as OlA—oleanolic acid and UrA—ursolic acid.

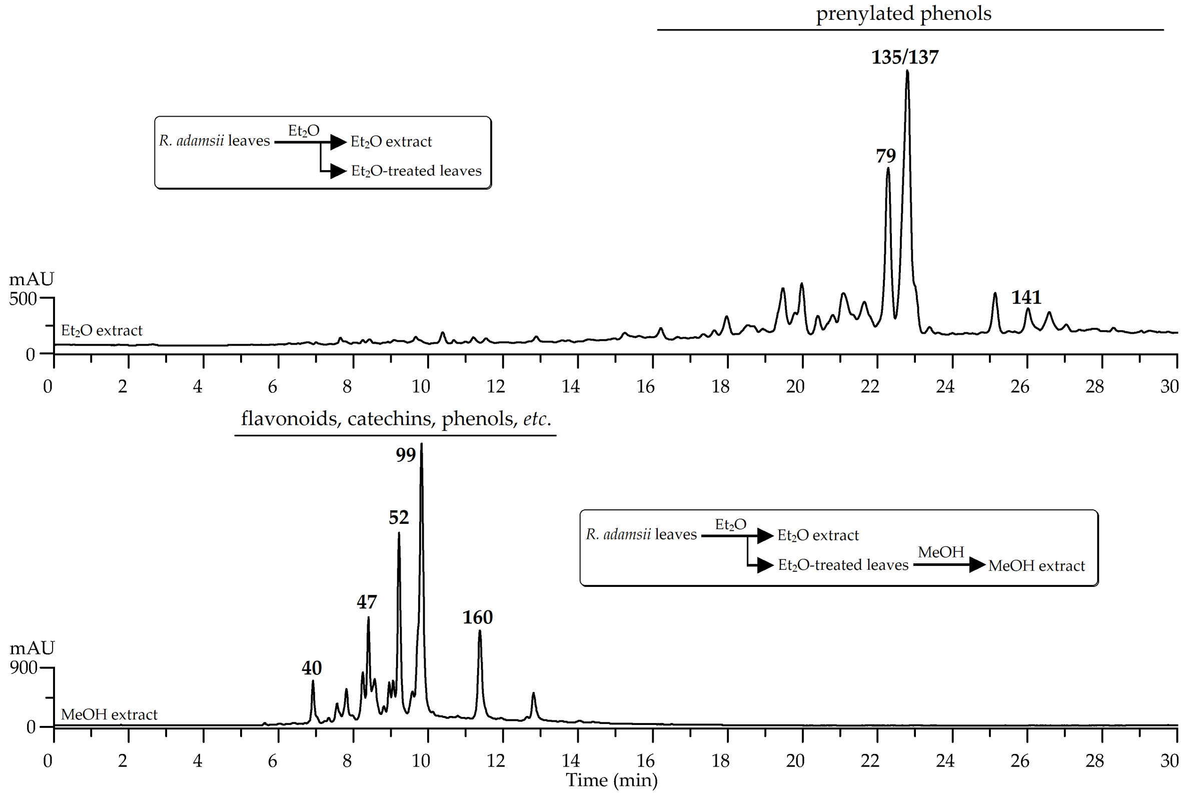

Figure S3. HPLC-PDA chromatograms of ether (Et_2O) and methanolic (MeOH) extracts of fresh *R. adamsii* leaves (January sample). The basic peaks numbered as described in Table 2. Sample preparation: Intact fresh *R. adamsii* leaves (5 g) were rinsed (10 min) three times in Et_2O (100 mL, each time; 20°C) and combined Et_2O-extract was concentrated in vacuum to dryness. The leaves of *R. adamsii* after Et_2O treatment were powdered and extracted with boiled MeOH twice (50 mL, each extraction) followed by concentration in vacuum to dryness to prepare MeOH-extract. Both extracts were dissolved in MeOH (*ca*. 5 mg in 1 mL), filtered through a 0.22-µm PTFE syringe filter before injection into the HPLC system. Chromatographic conditions used to separation of ethereal and methanolic extracts described in section 2.9 of Material and Methods.

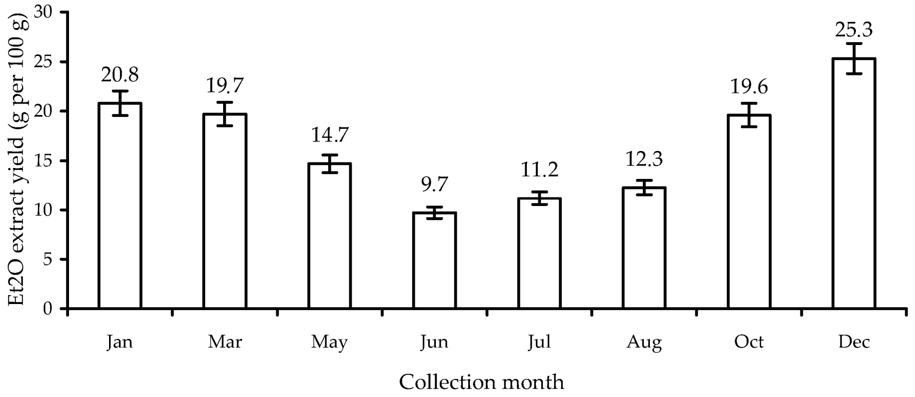

Figure S4. Yield of ether extract from *R. adamsii* leaves collected in various months. Sample preparation: Powdered *R. adamsii* leaves (1 g) were extracted three times with Et_2O (30 mL, each time; 20°C) and combined Et_2O-extract was concentrated in vacuum to dryness. The dry residue was weighted and the yield of ether extract was expressed as g per 100 g of dry leaves. All the analyses were carried out three times.
